# Neuronal Wiring Receptors Dprs and DIPs Are GPI Anchored and This Modification Contributes to Their Cell Surface Organization

**DOI:** 10.1523/ENEURO.0184-23.2023

**Published:** 2024-02-09

**Authors:** Meike Lobb-Rabe, Wioletta I. Nawrocka, Ruiling Zhang, James Ashley, Robert A. Carrillo, Engin Özkan

**Affiliations:** ^1^Department of Molecular Genetics and Cell Biology, The University of Chicago, Chicago, Illinois 60637; ^2^Program in Cell and Molecular Biology, The University of Chicago, Chicago, Illinois 60637; ^3^Neuroscience Institute, The University of Chicago, Chicago, Illinois 60637; ^4^Department of Biochemistry and Molecular Biology, The University of Chicago, Chicago, Illinois 60637; ^5^Institute for Biophysical Dynamics, The University of Chicago, Chicago, Illinois 60637; ^6^Committee on Development, Regeneration, and Stem Cell Biology, The University of Chicago, Chicago, Illinois 60637

**Keywords:** cell surface receptor, DIP, Dpr, *Drosophila melanogaster*, glycosylphosphatidylinositol anchor, motor neuron, muscle, surface localization, synapse

## Abstract

The *Drosophila* Dpr and DIP proteins belong to the immunoglobulin superfamily of cell surface proteins (CSPs). Their hetero- and homophilic interactions have been implicated in a variety of neuronal functions, including synaptic connectivity, cell survival, and axon fasciculation. However, the signaling pathways underlying these diverse functions are unknown. To gain insight into Dpr–DIP signaling, we sought to examine how these CSPs are associated with the membrane. Specifically, we asked whether Dprs and DIPs are integral membrane proteins or membrane anchored through the addition of glycosylphosphatidylinositol (GPI) linkage. We demonstrate that most Dprs and DIPs are GPI anchored to the membrane of insect cells and validate these findings for some family members in vivo using *Drosophila* larvae, where GPI anchor cleavage results in loss of surface labeling. Additionally, we show that GPI cleavage abrogates aggregation of insect cells expressing cognate Dpr–DIP partners. To test if the GPI anchor affects Dpr and DIP localization, we replaced it with a transmembrane domain and observed perturbation of subcellular localization on motor neurons and muscles. These data suggest that membrane anchoring of Dprs and DIPs through GPI linkage is required for localization and that Dpr–DIP intracellular signaling likely requires transmembrane coreceptors.

## Significance Statement

The Dpr/DIP families of cell surface receptors are important for controlling neuronal circuit assembly and synaptic/neuronal signaling, as demonstrated in the visual, neuromuscular, and olfactory systems in the fruit fly. Here, we show both in cultured cells and in vivo that they are GPI-anchored adhesion molecules and therefore cannot directly signal into cells, likely requiring coreceptors. Furthermore, we demonstrate that the GPI anchoring contributes to punctate pre- and postsynaptic organization in the *Drosophila* neuromuscular system.

## Introduction

Cell surface proteins (CSPs) allow a cell to interact with neighboring cells and the extracellular matrix and interpret chemical cues from its environment. CSPs can be attached to cells through hydrophobic transmembrane domains that traverse the lipid bilayer or via post-translational modifications that anchor the protein in the plasma membrane. One such modification is the addition of a glycosylphosphatidylinositol (GPI) anchor, which is covalently linked to the C terminus of the protein and embeds its hydrophobic acyl chains into the outer leaflet of the cell membrane (Ikezawa et al., 1976; Low and Finean, 1977; Ferguson and Williams, 1988). GPI anchors are attached at the ω site, typically a small amino acid, flanked upstream by an unstructured region and downstream by a stretch of hydrophobic residues (Eisenhaber et al., 1999). The GPI anchor is added in the ER lumen during protein synthesis, and the mature protein is trafficked to the cell membrane through the secretory pathway (Orlean and Menon, 2007). GPI-anchored proteins cannot signal into the cells directly, as the anchors do not physically reach into the cell interior. Thus, the interaction of GPI-linked proteins with intracellular signaling pathways likely requires engagement of transmembrane coreceptors able to transduce the signal inside the cell.

The human genome is predicted to encode at least 129 GPI-anchored proteins (UniProt Consortium, 2015). These include molecules implicated in a variety of functions in the nervous system including axon guidance and synaptic adhesion (reviewed in Um and Ko, 2017). Abrogation of GPI anchoring and defects in biosynthesis of GPI anchors have been implicated in various diseases including central nervous system disorders (Lukacs et al., 2019; Wu et al., 2020).

Computational tools to predict GPI-anchored proteins have been unreliable due, in part, to small training sets and similarity of features associated with GPI anchors and transmembrane domains. Compounding issues with predictions, the membrane-anchoring status of a particular protein cannot simply be deduced from its homology with other proteins; protein families may include members that can be transmembrane, GPI anchored, and secreted. For example, the semaphorin family of key axon guidance cues includes members that are secreted (classes 2 and 3), transmembrane (classes 1, 4, 5, 6), and GPI anchored (class 7; Kruger et al., 2005). Sema7A is GPI anchored and involved in axonal outgrowth (Pasterkamp et al., 2003), synaptic pruning (Uesaka et al., 2014), and integrin-dependent stimulation of immune cells (Suzuki et al., 2008). Ephrins, the membrane-tethered ligands for the receptor tyrosine kinases, Ephs, are divided into GPI-anchored ephrin-A and single-pass transmembrane ephrin-B classes and act by binding their cognate EphA and EphB receptors, respectively (Gale et al., 1996; Eph Nomenclature Committee, 1997). Interestingly, GPI-anchored ephrin-A5 has been shown to transmit an intracellular signal despite the lack of intracellular region; it partitions into caveolae-like microdomains and engages Fyn protein tyrosine kinase signaling upon interaction with an externally applied soluble form of EphA5 receptor (Davy et al., 1999).

The IgLONs, a five-member family of conserved mammalian CSPs, are GPI anchored and function in neurite outgrowth and synaptogenesis (Struyk et al., 1995; Hachisuka et al., 1996; Itoh et al., 2008; Sanz et al., 2015). IgLON-mediated signaling remains poorly understood although one member, Negr1, is cleaved by an ADAM-family protease and binds to an FGF receptor to stimulate dendritic arbor growth (Pischedda and Piccoli, 2016). The *Drosophila melanogaster* orthologs of the IgLONs, the Dpr–DIP family of CSPs (Cheng et al., 2019b), have been implicated in a variety of functions, including synapse specificity (Carrillo et al., 2015; Ashley et al., 2019; Courgeon and Desplan, 2019; Menon et al., 2019; Venkatasubramanian et al., 2019; C. Xu et al., 2019), axonal pathfinding and fasciculation (Barish et al., 2018; Bornstein et al., 2021; Lobb-Rabe et al., 2022), cell fate determination (Courgeon and Desplan, 2019), cell survival (Carrillo et al., 2015; S. Xu et al., 2018, 2022; Courgeon and Desplan, 2019; Menon et al., 2019), and behavior (Nakamura et al., 2002; C. Xu et al., 2019; Brovero et al., 2021). Structurally, the ectodomains of Dprs and DIPs consist of two and three immunoglobulin (Ig) domains, respectively (Özkan et al., 2013; Carrillo et al., 2015), similar to IgLONs which have three Ig domains and interact in a structurally similar manner to Dprs and DIPs (Cheng et al., 2019b; Ranaivoson et al., 2019; Venkannagari et al., 2020). However, how these proteins are linked to the cell membrane has not been determined. Here, we tested whether members of the *Drosophila* Dpr/DIP family are GPI anchored. We observed that most Dprs and DIPs have a GPI anchor; this was not expected as only a few family members are predicted to have GPI anchors. To bolster our hypothesis that these proteins are GPI anchored, we examined a subset of Dprs and DIPs in insect cells and live fly tissues. Treatment with GPI-specific phospholipase-C (PLC) causes shedding of Dprs and DIPs from the cell and tissue surface; this cleavage is GPI anchor dependent, as it is lost when the GPI anchor site is replaced with a transmembrane domain of a CD4 glycoprotein. Additionally, cleavage of GPI anchors also abolishes Dpr–DIP-mediated cell aggregation. Finally, replacing the GPI anchor with a transmembrane domain perturbs presynaptic and postsynaptic localization of these CSPs. Together these findings suggest that GPI anchors of Dpr/DIP contribute to their localization and function.

## Materials and Methods

### Sequence selection, molecular cloning, and generation of UAS transgenics

cDNAs of most of the full-length Dprs and DIPs used in the study were obtained from the Drosophila Genomics Research Center (DGRC). The remaining full-length sequences were generated through the extension of existing ectodomain constructs (Özkan et al., 2013). Among the DGRC clones, the sequence of Dpr15 required modification to remove an insertion within the Ig2 domain to preserve its structural integrity. The source, isoform, and identification number of each used sequence can be found in Extended Data Table 1-1. Sequences of Dprs and DIPs with an N-terminal V5 tag, inserted after a signal peptide, were cloned into a modified version of the pMT/BiP/V5-His A vector (Invitrogen) for expression in S2 *Drosophila* cells for PLC cleavage assay or into the pAcGP67 baculovirus transfer vector (BD Biosciences) for expression in Sf9 cells for cell flow cytometry and cell aggregation experiments. For cell aggregation, the constructs additionally included EGFP or mScarlet separated from the N-terminally V5-tagged Dprs or DIPs by the P2A sequence.

For the UAS-DIP-α-CD4 construct, the signal peptide and the first Ig domain of DIP-α were amplified from UAS-DIP-α-Myc (Ashley et al., 2019) and the second and third Ig domains, transmembrane domain, and C terminus were amplified from UAS-CD4-spGFP1-10 (Gordon and Scott, 2009). These were then combined into the NotI site of pUASTattB using NEBuilder HIFI Assembly. This construct was injected by GenetiVision (genetivision.com) and integrated into the attP2 landing site (BDSC 8622).

UAS-V5-Dpr10 was adapted from S. Xu et al. (2018), with the V5 tag inserted after amino acid 25. The V5-Dpr10 sequence was amplified from the UAS-V5-Dpr10 line and cloned into the NotI site of the pUASTattB plasmid using NEBuilder HIFI Assembly.

For the UAS-CD4-Dpr10 construct, the mCD8 signal peptide was amplified from Mhc-mCD8-GFP (Zito et al., 1999), a V5-tag oligo was ordered (IDT), the last two Ig domains of CD4 amplified from UAS-CD4-spGFP1-10 (Gordon and Scott, 2009), the C terminus of Dpr10 (amino acids 252–362) was amplified, and then all combined into the NotI site of pUASTattB using NEBuilder HIFI Assembly.

For the UAS-Dpr10-CD4 construct, the signal peptide, V5 tag, and N terminus of Dpr10 (amino acids 1–251) were amplified from UAS-V5-Dpr10, and the transmembrane domain and C terminus of CD4 were amplified from UAS-CD4-spGFP1-10 (Gordon and Scott, 2009). These were combined into the NotI site of pUASTattB using NEBuilder HIFI Assembly. The Dpr10 constructs were injected by GenetiVision (genetivision.com) and integrated into the VK20 landing site (BDSC 9738).

UAS-V5-Dpr19 was amplified from the pMT-V5-Dpr19 (see above) using Dpr19 specific primers and cloned into the NotI site of pUASTattB using NEBuilder HIFI Assembly. This construct was injected by BestGene (thebestgene.com) and integrated into the attP2 landing site (BDSC 8622).

**Table:** 

Primer name	Primer sequence	Construct	Description
Dip-α-pUAST_FOR	gaattcgttaacagatctgcATGCCAGT CTCGGCGAAAC	UAS-DIP-α-CD4	Forward DIP-α primer (beginning of ORF) including pUAST sequences for HIFI cloning
Dip-α-CD4_REV	CCTCCTCCCGGAATCACGACGTCCAGGA	UAS-DIP-α-CD4	Reverse DIP-α primer (after 1st Ig domain) including overlap sequence with CD4
CD4-Dip-α_FOR	GTGATTCCGttccagaaggcctccagcatagtc	UAS-DIP-α-CD4	Forward CD4 primer (starting with 3rd Ig domain) including overlap sequence with DIP-α
CD4-pUAST_REV	gtaccctcgagccgcttagcgccttcggtg	UAS-DIP-α-CD4	Reverse CD4 primer (C terminus) including overlap sequence with pUAST
F-pUAST-mCD8 (signal)	Gaattcgttaacagatctgcatggcctcaccgttgacc	UAS-CD4-Dpr10	Forward mCD8 primer (signal peptide) including pUAST sequences for HIFI cloning
R-mCD8 (signal) V5-tag	ggcttgccaaagattcggagttcgggtgc	UAS-CD4-Dpr10	Reverse mCD8 primer (signal peptide) including V5-tag overlap sequence
mCD8-V5-CD4	gaatctttggcaagccgatcccgaatc cactgctcggactggatagcaccggcggatcctccagcat	UAS-CD4-Dpr10	Synthesized DNA from IDT including overlap sequences with both mCD8 and CD4
F-V5tag-CD4 (Ig3)	Cggatcctccagcatagtctataagaaagaggggga	UAS-CD4-Dpr10	Forward CD4 primer (starting with 3rd Ig domain) including overlap with V5-tag sequence
R-CD4 (Ig4)-Dpr10 (TM)	CCCTGAAGtggctgcaccggggt	UAS-CD4-Dpr10	Reverse CD4 primer (after 4th Ig domain) including Dpr10 overlap sequence
F-CD4 (Ig4)-Dpr10 (TM)	gcagccaCTTCAGGGCGAGCGACC	UAS-CD4-Dpr10	Forward Dpr10 primer (after 2nd Ig domain) including CD4 overlap
R-Dpr10 (TM)-pUAST	gtaccctcgagccgcCTACCGTGC ATTCCTGCTGCC	UAS-CD4-Dpr10 (UAS-V5-Dpr10)	Reverse Dpr10 primer (C terminus) including pUAST overlap
F-pUAST-Dpr10 (1–19)	gaattcgttaacagatctgcATGTTG ACCCTGTGGACGG	UAS-Dpr10-CD4 (UAS-V5-Dpr10)	Forward Dpr10 primer (beginning of ORF) including overlap with pUAST
R-Dpr10-CD4 (TM)	gtggaccaGACATGGACGCGTATGCTGG	UAS-Dpr10-CD4	Reverse Dpr10 primer (after 2nd Ig domain) including overlap with CD4 transmembrane sequence
F-Dpr10-CD4 (TM)	GTCCATGTCtggtccaccccggtgca	UAS-Dpr10-CD4	Forward CD4 primer (after 4th Ig domain) including overlap with Dpr10 sequence
R-CD4 (TM)-pUAST	gtaccctcgagccgcttagcgccttcggtg	UAS-Dpr10-CD4	Reverse CD4 primer (C terminus) including overlap with pUAST sequence
Dpr19_V5-pUAST.For	gaattcgttaacagatctgcATGAAGT TATGCATATTACTGGCCGT	UAS-V5-Dpr19	Forward Dpr19 primer with BIP signal peptide (see above) including pUAST overlap
Dpr19_V5-pUAST.Rev	gtaccctcgagccgcTCAATGGTG ATGGTGATGATGACC	UAS-V5-Dpr19	Reverse Dpr19 primer (C terminus) including pUAST overlap

### Predictions of GPI anchors and transmembrane helices

Predictions of GPI linkage were carried out using PredGPI (Pierleoni et al., 2008) and NetGPI (Gíslason et al., 2021) tools available online. For predictions using PredGPI, the general model was used. Predictions of transmembrane regions were performed using Phobius (Käll et al., 2004) and DeepTMHMM (Hallgren et al., 2022). Before analysis in Phobius and DeepTMHMM, the sequences of signal peptides predicted with SignalP-6.0 (Teufel et al., 2022) were removed.

### *Drosophila* reagents, dissections, and immunohistochemistry

All flies were kept at 25°C except when otherwise noted. Crosses were set at medium density, and crawling third instar larvae were dissected as before (Wang et al., 2022) in phosphate-buffered saline (PBS, pH 7.4) on Sylgard dishes. Briefly, after fixing for 30 min in 4% paraformaldehyde, fillets were washed three times for 10 min in PBST (PBS + 0.05% Triton X-100, pH 7.4). Larval fillets were then blocked in 5% normal goat serum in PBST. Samples were incubated in primary antibodies diluted in block and overnight at 4°C, extensively washed in PBST, and incubated with secondary antibodies for 2 h at room temperature before the final washes in PBST. All washes and incubations occurred on nutators. Samples were then mounted in Vectashield (Vector Laboratories), and representative images were collected. HRP was used as a marker for neuronal membranes and DLG was used as a marker for postsynaptic membranes. Both sexes were used in this study.

The antibodies used for this study are the following:

**Table:** 

Antibodies	Source	Concentration
Chicken anti-V5	Bethyl Laboratories (A190–118A)	1:200
Mouse anti-V5	Thermo Fisher (R960-25)	1:200
Rabbit anti-V5	Cell Signaling Technology (D3H8Q)	1:10,000
Rabbit anti-DLG	Budnik Lab (PDZ2; Thomas et al., 1997)	1:40,000
Mouse anti-Myc	MilliporeSigma (05-724)	1:50
Rabbit anti-CD4	Novus Biologicals (NBP1-86143)	1:50
Mouse anti-α-Tubulin	MilliporeSigma (DM1A)	1:5,000
Goat anti-Mouse 488	Thermo Fisher (A11029)	1:500
Goat anti-Rabbit 488	Thermo Fisher (A11008)	1:500
Goat anti-Chicken 488	Thermo Fisher (A11039)	1:500
Goat anti-Rabbit 568	Thermo Fisher (A11036)	1:500
Goat anti-HRP 647	Jackson ImmunoResearch (123-605-021)	1:100
Goat anti-Mouse 680	Jackson ImmunoResearch (115-625-146)	1:5,000
Goat anti-Rabbit 790	Jackson ImmunoResearch (111-655-144)	1:5,000

The fly lines used for this study are the following:

**Table:** 

Genotype	Description	Source
*W1118*	White controls	Bloomington Drosophila Stock Center (BDSC)
*Mef2-GAL4*	Muscle GAL4 driver	Ranganayakulu, et al. (1998)
*A8-GAL4*	Is neuron GAL4 driver	Venkatasubramanian et al. (2019) and Wang et al. (2021)
*UAS-V5-Dpr19*	N-terminally V5-tagged Dpr19	This study (attP2/BDSC 8622)
*UAS-V5-Dpr10*	N-terminally V5-tagged Dpr10	This study (VK20/BDSC 9738)
*UAS-Dpr10-CD4*	Ig domains from Dpr10, and the TMD and C terminus of CD4	This study (VK20/BDSC 9738)
*UAS-CD4-Dpr10*	Replaced Dpr10 Ig domains with last two Ig domains of CD4, remainder of the protein from Dpr10	This study (VK20/BDSC 9738)
*UAS-Myc-DIP-α*	N-terminally Myc-tagged DIP-α	Gift from Larry Zipursky
*UAS-DIP-α-CD4*	First Ig domain of DIP-α added to the C terminus of CD4, including the final two Ig domains and TM domain of CD4	This study (attP2/BDSC 8622)

### PLC cleavage assay with S2 cells

S2 cells were obtained from the Drosophila Genomics Research Center and maintained in Schneider's Medium (Sigma S0146), supplemented with Insect Medium Supplement (Sigma I7267), and 90 U/ml penicillin plus 90 µg/ml streptomycin. Cells were maintained at room temperature. For experiments, confluent cells were split 1:2 into six-well plates and transfected the following day. Complete Schneider’s Media were mixed 2:1 with 250 µg/ml dimethyldioctadecyl-ammonium bromide (DDAB) and allowed to mix, and then 500 µl DNA was added for each well to be transfected (Han, 1996). Approximately 24 h after transfection, protein expression was induced by adding 1 mM copper sulfate (CuSO_4_). Three days later, 2 µl of 100 U/ml phosphatidylinositol-specific phospholipase C (PI-PLC, Life Technologies P-6466) was added to the treatment well, and cells were incubated for 4 h (protocol adapted from Butler et al., 1997).

To harvest cells, we collected and spun down 2 ml from each well at 500 × *g* for 5 min. The supernatant was collected and mixed with 6× loading buffer (375 mM Tris-Cl, pH 6.8, 9% SDS, 50% glycerol, 0.03% bromophenol blue, 9% β-mercaptoethanol) and boiled for 10 min. The cell pellet was washed in PBS and spun down as before. Next, cells were lysed using a buffer adapted from Bumgarner et al. (2005), consisting of 50 mM Tris-HCl, pH 8.0, 150 mM NaCl, 1% Triton X-100, 5 mM EDTA, and protease inhibitors (one tablet per 50 ml; Pierce A32955). Tubes were incubated on a nutator at 4°C for 30 min and then at 37°C for 30 min. Tubes were centrifuged at 17,000 × *g* at room temperature for 15 min and mixed with 6× loading buffer.

Samples were then run on 12% SDS-polyacrylamide gels using the TGX FastCast system (Bio-Rad 1610175). The samples were transferred overnight onto a nitrocellulose membrane (Bio-Rad 1620115) at a constant current of 40 mA and blocked for 1 h in 1% (w/v) casein block in PBS. Staining with primary and secondary antibodies was performed for 2 h at room temperature with slight agitation and included washes in PBST after each incubation. Westerns were imaged on a LI-COR Odyssey imager.

For quantification, ROIs (regions of interest) were drawn around bands on the Western—the same size ROI was used from control and +PLC conditions. The raw integrated density (RawIntDen), a measure of the summed pixel intensity within the ROI, was obtained with ImageJ FIJI (Schindelin et al., 2012). This quantification is shown as a before and after graph (Extended Data Fig. 2-1) generated using GraphPad Prism.

### Flow cytometry

Sf9 cell cultures at the density of 2 × 10^6^ cells/ml were placed in six-well plates, 3.5 ml per well. The cells were infected with baculoviruses encoding wild-type Dprs and DIPs with an N-terminal V5 tag inserted after the GP64 signal sequence, and variants in which the GPI signal was replaced by the transmembrane domain (TMD) of rat neurexin-1 (amino acids 1454–1479; UniProtKB/Swiss-Prot: Q63372.3). Infected cultures were incubated for 48 h on an orbital shaker at 125 rpm (Thermo MaxQ 430) at 28°C. We transferred 0.6 ml of each culture to a 24-well plate. For GPI cleavage, 4 µl of PI-PLC per well was used. We added 4 µl of PI-PLC storage buffer (20 mM Tris-HCl, pH 7.5, 1 mM EDTA, 0.01% sodium azide, 50% glycerol) to control samples. Each sample was prepared in five replicates. Treatment was carried out for 3 h on an orbital shaker at 125 rpm at 28°C. Cultures were spun down for 1 min at 500 × *g*. The cell pellets were resuspended in 300 µl of cold PBS, pH 7.4, with 1% BSA. A total of 100 µl of cell suspensions were transferred to a U-shaped 96-well plate. We used 4 µl of anti-V5-AF647 antibody (R&D Systems FAB8926R) for staining cells for 20 min on a shaker at 500 rpm (Thermo Microplate Shaker 11-676-337) at 4°C. Cultures were spun down for 1 min at 500 × *g*, at 4°C, and washed three times with 200 µl of PBS, pH 7.4, with 1% (w/v) BSA. Cells were analyzed using a BD Accuri C6 flow cytometer. Fifteen thousand events were recorded per sample. The results were analyzed using FlowJo Software v10.8.1 (BD Life Sciences).

### Cell aggregation assay with Sf9 cells

Sf9 cultures at the density of 2 × 10^6^ cells/ml were placed in six-well plates, 3 ml per well, and infected with baculoviruses encoding (N- to C-terminal) mScarlet, P2A sequence (ATNFSLLKQAGDVEENPGP), GP64 signal peptide, and V5-tagged Dpr; or EGFP, P2A sequence, GP64 signal peptide, and V5-tagged DIP. The P2A peptide results in ribosome skipping (Szymczak-Workman et al., 2012) and separate polypeptide chains for mScarlet/EGFP and V5-tagged Dpr/DIPs. This construct design was chosen to leave unmodified C termini for Dprs and DIPs, as that would be important for testing for a C-terminal GPI anchoring signal. Infected cultures were incubated for 48 h on an orbital shaker at 125 rpm at 28°C. Expression of V5-tagged Dprs and DIPs was confirmed using Western blots with the anti-V5-AF647 antibody. Cultures were diluted 1:15 in Sf9 complete media (Invitrogen Sf-900 III SFM, with 10% FBS, 2 mM L-glutamine, and 20 µg/ml gentamicin). A total of 200 µl of cultures expressing Dprs were mixed with 200 µl of cultures expressing DIPs in 24-well plates. The control samples included 200 µl of cultures expressing individual Dprs or DIPs mixed with 200 µl of noninfected Sf9 cultures. Samples were prepared in triplicates for each Dpr–DIP pair and each control culture expressing individual Dprs and DIPs. Cultures were left to aggregate for 30 min on an orbital shaker at 125 rpm at 28°C. For GPI cleavage, 4 µl of PI-PLC per well was used. We added 4 µl of PI-PLC storage buffer to control samples. Treatment was carried out for 1 h on an orbital shaker at 125 rpm at 28°C. Cultures were imaged in 24-well plates at 5× magnification. Three images were collected for every well. Cell aggregation was quantified using the cell aggregation index, defined as the percentage of the total area occupied by cells that is composed of aggregates (Boucard et al., 2012). The area occupied by cells and aggregates was determined using the “Analyze particles” function in Fiji. Aggregates were defined as particles with areas of at least 2,900 µm^2^.

### Tissue PLC experiment

Larvae were dissected and processed as before (Carrillo et al., 2015). Briefly, larvae were filleted, rinsed in PBS, and incubated while shaking for 1 h in 1 ml of PBS with or without 1 µl of PI-PLC. Larvae were washed with PBS, fixed, and stained as above with a slight alteration: detergent was only used after staining for Dpr/DIP protein tags (Ashley et al., 2019). After extensive washing in PBST, anti-DLG was diluted in PBST and incubated with larvae for 2 h at room temperature. The subsequent washing and secondary antibody staining was as described above. The staining procedure for PLC-treated and untreated preparations was carried out in the same tube so that conditions were identical. Three muscle 4 Ib arbors were imaged per animal.

For quantification, comparisons were made on samples processed and imaged on the same day. Also, images were collected from each experiment using identical imaging parameters and analyzed with ImageJ FIJI (Schindelin et al., 2012). ROIs were drawn around the three terminal Ib boutons and the pixel sum was measured. The signal of Dpr/DIP was normalized to DLG signal to control for any differences in staining conditions between replicates and because DLG should not be affected by PLC treatment. These values are reported as “Relative Pixel Sum” representing Tag/DLG pixel sum.

### Protein localization in tissue

Because the GAL4/UAS system produces significant overexpression at 25°C, crosses were maintained at 18°C to dampen protein expression levels. Third instar larvae were dissected, incubated in respective antibodies, and mounted as described above. Detergents were omitted during the staining process to label only those proteins on the cell surface at the time of fixation. AiryScan images were collected on a Zeiss LSM880 confocal microscope using a Plan-Apo 63× objective at a zoom factor of 3 and acquired with bidirectional scanning and 8 bit image depth. Laser lines at 488 nm for V5/Myc/CD4 and 633 nm for HRP were used. Images were processed with AiryScan Processing at AUTO level.

Once images were collected, *z*-stack projections were generated, and a uniform threshold was set to convert each image to binary representation of the surface protein stain. The same threshold was used for all images. This image was then used to count particles using the “Analyze Particles” function. For presynaptic localization, an ROI was drawn around the three terminal boutons of each arbor and particles were measured. For postsynaptic localization, particles of the entire image were counted. These manipulations were performed using ImageJ FIJI (Schindelin et al., 2012).

### Statistical analysis

For tissue PLC experiments and tissue localization experiments, two-tailed Student's *t* test was used to determine statistical significance (GraphPad Prism 8). Cell aggregation experiments were evaluated using one-way ANOVA (GraphPad Prism 9).

### Tissue and cell imaging protocol

All in vivo images (except for Fig. 6; see Protein localization in tissue above) were obtained on a Zeiss LSM800 confocal microscope with a 40× Plan-Neofluar 1.3NA objective or 63× Plan-Apo 1.4NA objective. All images of Sf9 cells were obtained using a Leica THUNDER Imager 3D Cell Culture with Leica N Plan 5×/0.12 PH0 objective.

## Results

### Some Dprs and DIPs are predicted to be GPI anchored

**Figure 1.**
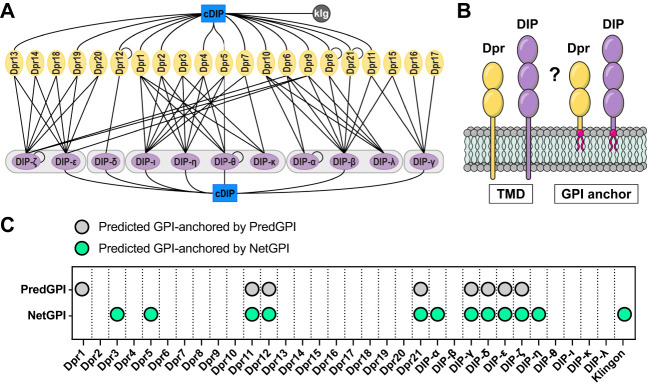
GPI anchoring predictions for the Dpr and DIP families of CSPs. ***A***, Dpr–DIP interactome. Lines indicate interactions determined in previously published in vitro studies. cDIP, common Dpr and DIP-interacting protein; Klg, Klingon. ***B***, Two potential membrane-anchoring modes of Dprs and DIPs. TMD, transmembrane domain; GPI, glycosylphosphatidylinositol. ***C***, GPI anchor predictions using PredGPI and NetGPI. Positive predictions are shown as green for NetGPI and gray for PredGPI. The isoforms we used for predictions are tabulated in Extended Data Table 1-1, and more details for the prediction results are in Extended Data Table 1-2 and Figure 1-1.

The Dprs and DIPs bind selectively with one another via their ectodomains, forming an elaborate network of homo- and heterophilic interactions (Fig. 1*A*). Their multifunctional roles in neural circuit development partially rely on their abilities to act as cell adhesion molecules. However, whether and how Dprs and DIPs signal intracellularly have not been examined. Determining if they are transmembrane (TM) or GPI-anchored proteins would shed light on their potential signaling mechanisms.

10.1523/ENEURO.0184-23.2023.f1-1 Figure 1-1. **Transmembrane region and GPI anchoring predictions.** (A) Positive predictions for the presence of transmembrane domains are shown as green circles for DeepTMHMM and gray circles for Phobius. (B) Positive GPI anchoring predictions by at least one program are shown as green circles, and positive TM predictions by at least one program are shown as gray circles. Download Figure 1-1, TIF file.

10.1523/ENEURO.0184-23.2023.t1-1 Table 1-1. **Selection of sequences used in our analyses.** X under isoform indicates the exact sequence for the predicted isoform does not exist in Flybase. Extended ECD indicates that the constructs were created by extending existing cDNA from our ectodomain expression library (Özkan et al., 2013). Download Table 1-1, XLS file.

10.1523/ENEURO.0184-23.2023.t1-2 Table 1-2. **Predictions by GPI detection software.** PredGPI reports a score called the *specificity index*, which classifies GPI anchoring as highly probable (>99.9%), probable (99.5–99.9%), lowly probable (99.0–99.5%), or not probable (<99%) (Pierleoni et al., 2008). NetGPI reports a Likelihood value for the GPI-anchor site (w) when GPI anchoring is predicted, and the likelihood of no GPI anchoring in the opposite case. Higher values indicate higher confidence in the reported ω site or lack of it (Gíslason et al., 2021). Download Table 1-2, XLS file.

To gain insight into how Dprs and DIPs associate with the cell membrane, we used two transmembrane region prediction tools, Phobius (Käll et al., 2004) and DeepTMHMM (Hallgren et al., 2022). We first removed N-terminal signal peptides recognized by SignalP-6.0 (Teufel et al., 2022) to avoid their classification as TM domains, which is an inherent challenge for prediction tools due to the hydrophobic nature of both types of sequences. Phobius predicted 15 of the Dprs and DIPs and Klingon, a *Drosophila* immunoglobulin superfamily (IgSF) protein previously demonstrated to be GPI anchored (Butler et al., 1997), to have C-terminal TM regions while DeepTMHMM indicated only Dpr9 as a TM protein (Extended Data Fig. 1-1).

Considering that the mammalian homologs of Dprs and DIPs are anchored to the membrane via GPI modification (Itoh et al., 2008), we hypothesized that the same may be true for Dprs and DIPs that were not predicted to be transmembrane proteins (Fig. 1*B*). We used two GPI signal prediction tools, PredGPI (Pierleoni et al., 2008) and NetGPI (Gíslason et al., 2021). PredGPI is built using a hidden Markov model (HMM) and a support vector machine (SVM), trained on experimentally validated GPI-anchored proteins, and produces minimal false positives while correctly identifying 89% of known GPI-containing proteins. The output of the PredGPI prediction includes the most likely position of the ω site—the residue to which the lipid anchor is attached and the “specificity” index expressing a measure of probability of the protein to be GPI anchored. The recently released NetGPI uses recurrent neural networks to predict GPI anchoring and reports the most likely position of the ω site or absence of one, with a likelihood value for either possibility. NetGPI was reported to slightly outperform PredGPI (Gíslason et al., 2021).

We examined all 32 members of the Dpr/DIP family and Klingon with PredGPI and NetGPI, and the majority of these CSPs were predicted to lack GPI anchors (Fig. 1*C* and Extended Data Table 1-2). PredGPI indicated eight Dprs and DIPs (25%) as likely GPI linked (specificity index above 99%; Extended Data Table 1-2) and Klingon as not GPI anchored as it fell under the threshold for positivity with its specificity index of 98.5% (Extended Data Table 1-2). NetGPI predicted 11 Dprs and DIPs (34%) to be GPI anchored and correctly classified Klingon. Among the proteins predicted to have a GPI modification by NetGPI, 67% were also predicted by PredGPI. Dpr3, Dpr5, and DIP-η were classified by NetGPI as likely GPI anchored but obtained very low specificity index values of 37.3, 16.0, and 1.0%, respectively, with PredGPI.

Overall, 12 Dprs and DIPs were predicted to be GPI anchored by either PredGPI or NetGPI (Extended Data Fig. 1-2). Fifteen Dprs and DIPs were predicted to have TM helices, and five of these CSPs were also predicted to have a GPI anchor (Dpr5, Dpr11, DIP-γ, DIP-ζ, and DIP-η). Ten Dprs and DIPs were not predicted to be GPI linked nor to contain a TM helix (Dpr4, Dpr6, Dpr10, Dpr13, Dpr15, Dpr16, Dpr17, DIP-θ, DIP-ι). Thus, using prediction tools alone did not unequivocally classify how these CSPs are anchored to the cell.

### Dprs and DIPs are anchored to the membrane via glycophosphatidyl linkages

**Figure 2.**
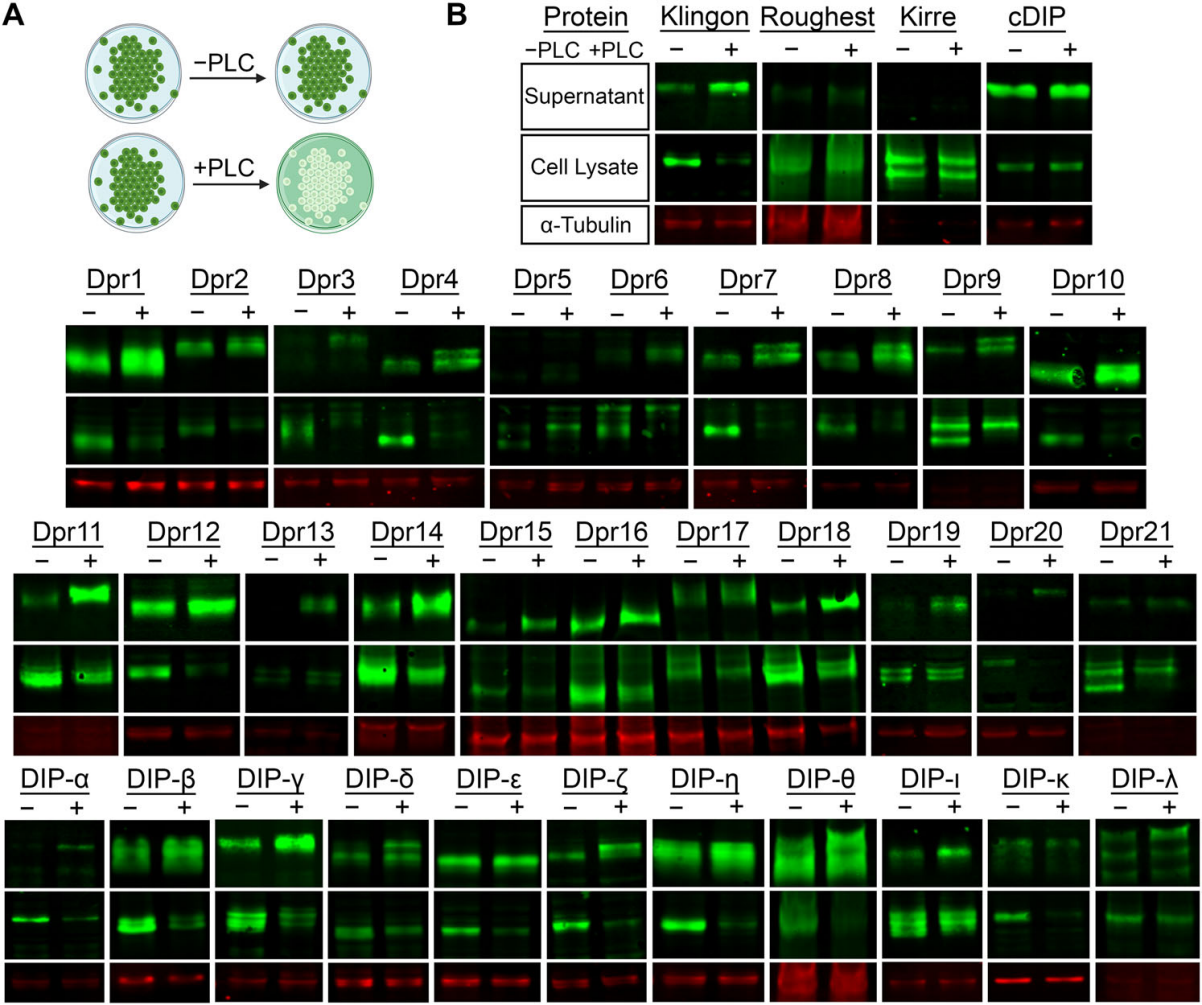
PLC treatment cleaves Dprs and DIPs from S2 cells, as observed by Western blotting. ***A***, Schematic for PLC cleavage assay on S2 cells, when a CSP is GPI anchored. Green color indicates where the GPI-anchored protein is (on cells or in culture media). ***B***, Western blots of the supernatant fraction, cell lysate, and Tubulin-α loading control for with (+) and without (−) PLC treatment for all members of Dpr/DIP subfamilies. See key in the top row for ***B***. Quantitation for the bands (including replicates) is shown in Extended Data Figure 2-1. The full amino acid sequences for the V5-tagged constructs are tabulated in Extended Data Table 2-1.

Because of limitations in GPI predictions, such as the lack of a consensus GPI motif, we hypothesized that the predictors underestimated the number of Dprs and DIPs that are GPI modified. To complement the prediction tools and examine the membrane anchoring mechanism(s) of all Dprs and DIPs, we set up an S2 cell culture pipeline for proteins with an N-terminal V5 tag inserted after the signal peptide (see Extended Data Table 1-1 and Materials and Methods section for details). Duplicate S2 cultures were established for each CSP, and one culture was treated with the GPI-cleaving enzyme phospholipase C (PLC). Supernatant and cell fractions were collected and used for Western blot analyses to determine if the CSP was cleaved by PLC. The GPI-anchored protein Klingon was used as the positive control and the secreted cDIP and transmembrane IgSF proteins Roughest (Rst) and Kirre served as negative controls (Ramos et al., 1993; Butler et al., 1997; Ruiz-Gómez et al., 2000; Carrillo et al., 2015). If the CSPs are GPI anchored, we expect an increase of protein in the supernatant and a concomitant decrease in the cell fraction after PLC treatment (Fig. 2*A*). Remarkably, most Dprs and DIPs displayed these trends, suggesting that a majority of Dprs and DIPs may be GPI anchored (Fig. 2*B*). We observed differences between samples with some CSPs cleaved more readily than others (i.e., more CSP in the supernatant or less CSP in the cell lysate after PLC treatment; Extended Data Fig. 2-1). However, even our positive control, Klingon, was not completely cleaved (Fig. 2*B*), as previously observed (Butler et al., 1997). These observations may be due to incomplete GPI processing and trafficking, incomplete access of the PLC to a subset of CSPs, chemical heterogeneities in GPI anchors, insufficient amounts of PLC used in the assay, or variations in CSP expression levels. Some of the CSPs appear as doublets in “−PLC” cell lysates. PLC treatment releases the lower band into media, as observed before in other GPI-anchored proteins (Moran et al., 1991), where the upper band was shown to represent full-length protein, not processed to add a GPI anchor and not yet transported to cell surface. Given the limitations of the experimental setup, we cannot definitively state the source for the observed doublets on gels.

10.1523/ENEURO.0184-23.2023.f2-1 Figure 2-1. **Quantification of western blots of PLC treated Dprs and DIPs from S2 cells.** (A) Raw integrated density values of western blots from experiments examining the supernatant fraction of Dprs and DIPs expressed in S2 cells (see Figure 2 for example blots). Black circles are values obtained from control samples that were not treated with PLC, while magenta squares are samples that were treated with PLC. Values that come from one experiment are linked with a black line. The protein examined is denoted on the *x*-axis. (B) Depicts the same experimental quantification but for the cell fraction rather than the supernatant. Download Figure 2-1, TIF file.

10.1523/ENEURO.0184-23.2023.t2-1 Table 2-1. **Protein sequences of open reading frames used in the PLC/SDS-PAGE assay in Figure 2.** The *Drosophila* BiP signal peptide is colored purple, and the V5-tag is colored green. The sequences of Dprs, DIPs, and the control proteins are in bold; their signal peptides have been removed. The remaining are linkers. Download Table 2-1, XLS file.

**Figure 3.**
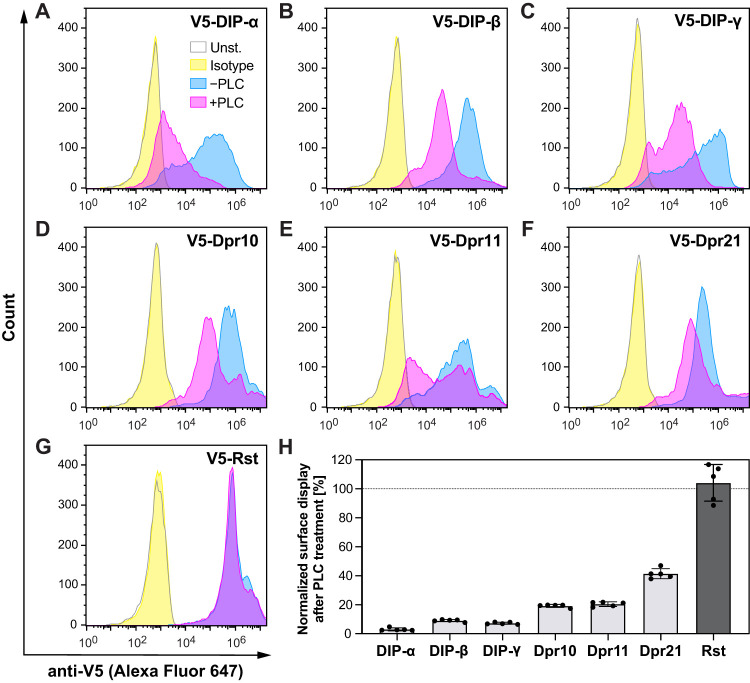
Surface display of Dprs and DIPs is reduced by PLC treatment, as observed by flow cytometry. ***A–G***, Histograms showing fluorescence levels of baculovirus-infected, unstained Sf9 cells (gray outline); cells infected and stained with anti-V5-Alexa Fluor 647 antibody (blue); cells infected, treated with PLC; and stained with anti-V5-Alexa Fluor 647 antibody (magenta); and cells infected and stained with rabbit IgG isotype Alexa Fluor 647 antibody (yellow). Rst, Roughest. ***H***, Remaining levels of wild-type and transmembrane (TMD) versions of Dprs and DIPs on Sf9 cell surface following PLC treatment, normalized as the ratio of median fluorescence intensity (MFI) for PLC-treated and not treated cells after subtraction of the background MFI value of unstained cells. Representative histogram plots for TMD variants are shown in Extended Data Figure 3-1.

We next used flow cytometry as an orthogonal method to demonstrate the GPI anchoring of Dprs and DIPs. We expressed the same, N-terminally V5-tagged constructs of three Dprs and three DIPs in Sf9 cells using baculoviral infection. The cultures expressing individual Dprs and DIPs, as well as the negative control, Rst, were split into two samples: one was treated with PLC, and the other served as an untreated control. Both samples were stained with fluorescent antibodies against V5 tag, and the relative levels of proteins on the surface of PLC-treated and untreated cells were assessed using a flow cytometer. All DIPs tested, DIP-α, DIP-β, and DIP-γ, showed a significant decrease in protein levels on the surface of Sf9 cells after PLC treatment (Fig. 3*A–C*). Similarly, all Dprs tested, Dpr10, Dpr11, and Dpr21, were mostly cleaved off the cell surface by PLC (Fig. 3*D–F*). In contrast, the cell surface level of Rst or variants of Dprs and DIPs with GPI signal replaced with the transmembrane domain of rat neurexin-1 remained unaffected by PLC cleavage (Fig. 3*G*,*H*, Extended Data Fig. 3-1), as expected for a transmembrane protein. The variable extent of cleavage between all tested Dprs and DIPs may be explained by the potentially different accessibility of the GPI cleavage site for each of the proteins. The results obtained using flow cytometry corroborate the observations made using Western blot analyses. Together, these findings demonstrate that most Dprs and DIPs are GPI anchored.

10.1523/ENEURO.0184-23.2023.f3-1 Figure 3-1. **Surface display of transmembrane versions of Dprs and DIPs is not affected by PLC treatment, as observed by flow cytometry.** (A-F) Histograms showing fluorescence levels of baculovirus-infected, unstained Sf9 cells (gray), cells infected and stained with anti-V5-Alexa Fluor 647 antibody (blue), cells infected, treated with PLC and stained with anti-V5-Alexa Fluor 647 antibody (magenta), and cells infected and stained with rabbit IgG isotype Alexa Fluor 647 antibody (yellow). TMD: transmembrane domain from rat Neurexin-1. Download Figure 3-1, TIF file.

### GPI anchor cleavage eliminates Dpr–DIP-mediated cell aggregation

Dprs and DIPs are thought to mediate cell adhesive interactions to accomplish many of their roles in the nervous system (S. Xu et al., 2022). A powerful in vitro approach to study cell adhesion interactions is the cell aggregation assay (Yoshida and Takeichi, 1982; Kumari and Varadaraj, 2009; Honig and Shapiro, 2020). Here, we combined cell aggregation and PLC assays to test whether cleavage of GPI anchors abrogates cell adhesion mediated by Dprs and DIPs.

For our combined aggregation/PLC assay, we used Sf9 cells infected with baculoviruses encoding mScarlet or EGFP followed by a P2A peptide and an open-reading frame to include the GP64 signal sequence, an N-terminal V5 tag, and the Dpr or DIP. We confirmed the expression of Dprs and DIPs using Western blots with an anti-V5 antibody (Extended Data Fig. 4-1*A*). Cultures expressing individual Dprs with mScarlet were mixed with cultures expressing cognate DIP partners and EGFP. We predicted that, if Dprs and DIPs are GPI-anchored cell adhesion molecules, Dpr–DIP interactions would lead to aggregation between the respective cells, and application of PLC would break up the aggregates.

Levels of clustering observed in cell aggregation assays are a function of (1) CSP expression levels, (2) CSPs' affinities and dissociation kinetics, (3) competing *cis* interactions, and (4) experimental conditions, such as mixing speeds, incubation, and washing times. While we can control (4), the others are different for every CSP pair, and therefore cell cluster size and extent of aggregation are different across different pairs of CSPs (Foty and Steinberg, 2005; Katsamba et al., 2009). However, since −PLC and +PLC samples for a given Dpr–DIP pair are split from the same population of transfected cells, our assay comparing Dpr–DIP-mediated aggregation with and without PLC treatment do not suffer from any of the variations in aggregation mentioned above.

**Figure 4.**
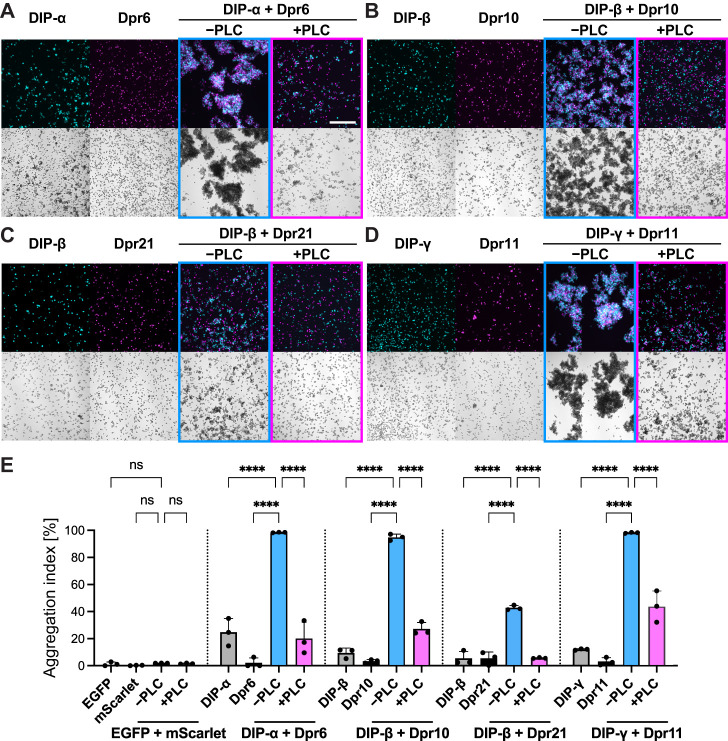
PLC cleavage eliminates Dpr–DIP-mediated cell aggregation. ***A–D***, Cell aggregation experiments with Sf9 cells. Controls included cultures expressing either DIP or Dpr (first two panels from the left) and cultures expressing DIP or Dpr mixed together but not treated with PLC (−PLC). Cell aggregation was abolished when PLC was added to the mixed cultures 30 min after aggregation (+PLC). Scale bar, 500 µm. ***E***, Cell aggregation index for the samples shown in ***A–D*** and the EGFP/mScarlet control; ****p < 0.0001; one-way ANOVA. See Extended Data Figure 4-1*A* for expression levels of each protein tested, and Extended Data Figure 4-1*B* for the aggregation assay for the EGFP/mScarlet only negative control.

Among the four cognate pairs of Dprs and DIPs tested, DIP-α and Dpr6, DIP-β and Dpr10, and DIP-γ and Dpr11 induced robust aggregation of Sf9 cells (Fig. 4*A–E*). DIP-β and Dpr21-expressing cells aggregated significantly less, which was unexpected considering their relatively high affinity compared with other Dpr–DIP pairs (Cosmanescu et al., 2018), and that both DIP-β and Dpr21 were expressed at considerable levels (Extended Data Fig. 4-1*A*). One reason for the reduced aggregation may be weak homophilic Dpr21 interactions (*K_D_* ∼ 50 µM) on the same cell that may prevent efficient heterophilic interactions with DIP-β between cells, as homophilic and heterophilic complexes use the same interfaces and cannot coexist (Cosmanescu et al., 2018; Cheng et al., 2019a). Nonetheless, the addition of PLC either significantly reduced or abolished cell aggregation for all four pairs of Dprs and DIPs (Fig. 4*E*). These results demonstrate that Dpr–DIP interactions instruct cell adhesion and suggest that Dprs and DIPs are anchored to the cell surface membrane via GPI modifications.

10.1523/ENEURO.0184-23.2023.f4-1 Figure 4-1. (A) Expression of N-terminally V5-tagged Dprs and DIPs in Sf9 cells used in cell aggregation assays. Sf9 cell pellets were solubilized and used in western blot with anti-V5-Alexa Fluor 647 antibody. L, molecular weight ladder. Expected molecular weights of proteins before N-linked glycosylation: DIP-α: 58.7 kDa; Dpr6: 41.9 kDa; DIP-γ: 45.7 kDa; Dpr11: 36.2 kDa; DIP-β: 53.1 kDa; Dpr10: 40.7 kDa; Dpr21: 31.7 kDa. Dprs and DIPs are N-glycosylated to various extent. (B) Negative control for cell aggregation experiments. Cell aggregation assay was performed with Sf9 cells infected with baculoviruses encoding only intracellular fluorescent proteins, EGFP and mScarlet. Scale bar, 500 μm. No aggregation was observed. Download Figure 4-1, TIF file.

### Dpr and DIP proteins are GPI anchored in vivo

Having demonstrated that most Dprs and DIPs appear to be GPI anchored in vitro, we hypothesized that the same modification occurs in fly tissue. To test this, we expressed tagged variants of a subset of Dprs and DIPs in the neuromuscular circuit using the GAL4-UAS system. The larval *Drosophila* neuromuscular junction (NMJ) is an excellent system to examine CSPs, as the circuit is well characterized and easily imaged. Moreover, Dprs and DIPs are endogenously expressed at the larval NMJ (Wang et al., 2022), and several Dprs and DIPs are implicated in NMJ development. DIP-α and Dpr10 are known to function in motor axon pathfinding and innervation (Ashley et al., 2019; Lobb-Rabe et al., 2022), and DIP-γ and Dpr11 regulate synaptic growth through the BMP pathway (Carrillo et al., 2015).

**Figure 5.**
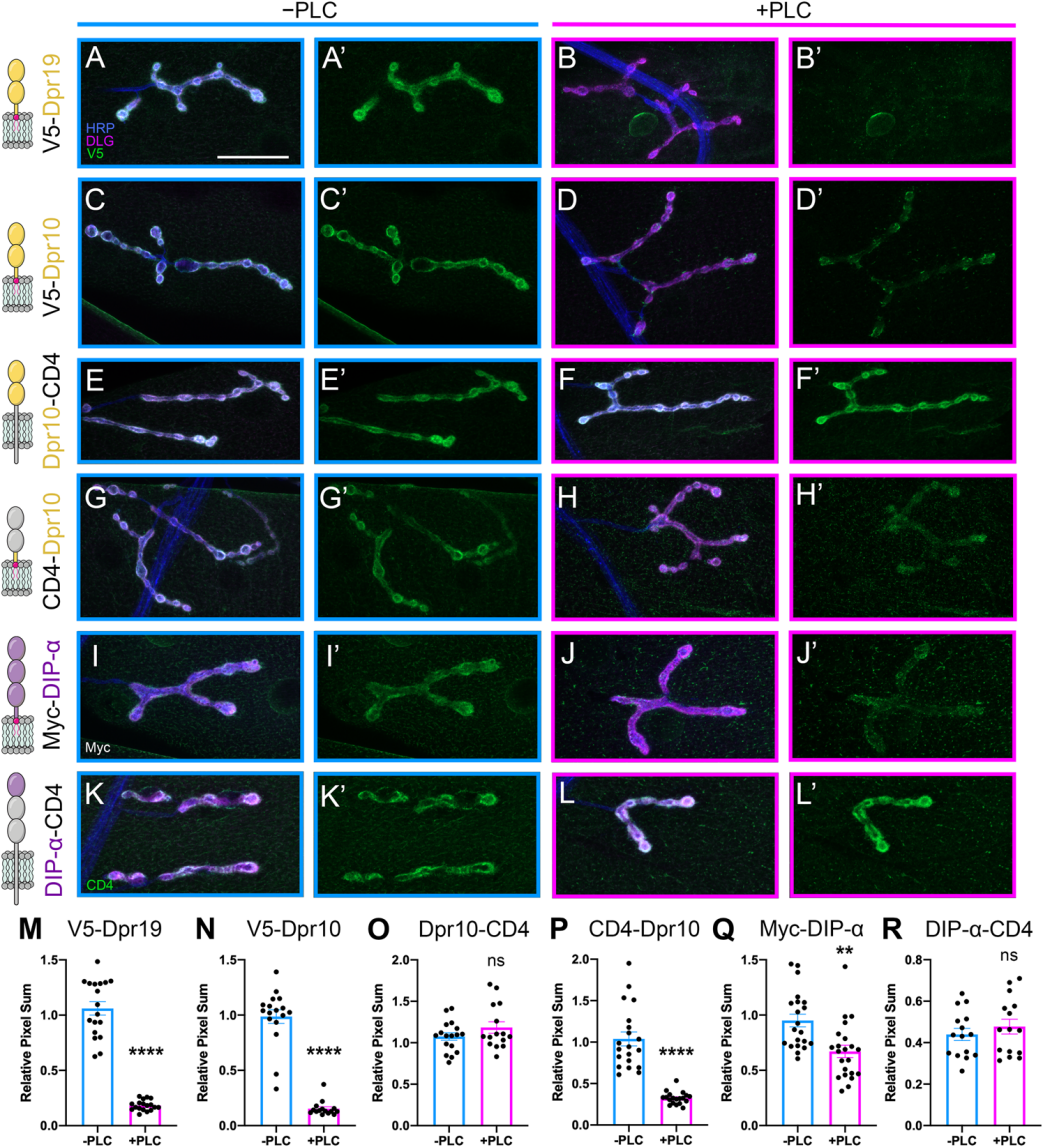
Dpr and DIP proteins are GPI anchored in vivo. ***A–L***′, Tagged Dprs and DIPs were expressed in muscles, and dissected larvae were treated with PLC and compared with nontreated controls. Cartoon presentations of tagged Dprs and DIPs and their domains are depicted in left column. Surface localized proteins are shown in green, neuronal tissue in blue (HRP), and postsynaptic membrane in magenta (DLG). ***A***, ***B***, PLC efficiently reduced surface labeling of V5 on muscles expressing V5-Dpr19 (*n *= 18/6 vs *n *= 18/6). ***C***, ***D***, PLC efficiently reduced surface labeling of V5 on muscles expressing V5-Dpr10 (*n *= 12/6 vs *n *= 10/6). ***E***, ***F***, PLC failed to reduce labeling of V5 in muscles expressing Dpr10-CD4 chimeras (*n *= 17/6 vs *n *= 15/6). ***G***, ***H***, PLC efficiently reduced surface labeling of V5 in muscles expressing CD4-Dpr10 chimeras (*n *= 20/9 vs *n *= 19/9). ***I***, ***J***, PLC reduced surface labeling of Myc on muscles expressing Myc-DIP-α (*n *= 21/9 vs *n *= 22/9). ***K***, ***L***, PLC failed to reduce muscle labeling of CD4 on muscles expressing DIP-α-CD4 chimeras (*n *= 15/6 vs *n *= 15/6). ***M–R*** Quantification of experiments shown in ***A***–***L′***. Number of *n* reported as *n *= *a*/*b* where *a* is the segments and *b* the animals for −PLC versus +PLC treatment. Scale bar, 50 µm. **p = 0.0097, ****p < 0.0001; *t* test.

To achieve high protein levels in a cell type that would allow for easy visualization of Dprs and DIPs on the cell surface, we expressed proteins in muscles using *Mef2-GAL4* and omitted detergents in the initial steps of our staining protocol. Muscles are highly stereotyped, multinucleated cells that are easily imaged. In third instar larvae, V5-Dpr19 localized to the subsynaptic reticulum (SSR), a network of postsynaptic membrane folds surrounding the innervating presynaptic boutons (Fig. 5*A*,*B*). After incubation with PLC, nearly all surface V5 signal was lost, indicating that Dpr19 is GPI anchored in vivo (Fig. 5*M*). Similarly, V5-Dpr10 was localized to the SSR and PLC treatment significantly decreased the V5 signal (Fig. 5*C*,*D*,*N*). However, significant Dpr10 remained on the muscle surface in puncta surrounding boutons (Fig. 5*D*,*D*′), suggesting that a population of Dpr10 was inaccessible to PLC or prevented from diffusing away by interacting with other CSPs at these sites. This observation matches the S2 and Sf9 insect cell data showing incomplete cleavage (Figs. 2–4). To confirm that loss of the cell surface signal was due to cleavage of the GPI anchor, we generated chimeric membrane-tethered Dpr10 proteins by replacing the C terminus of Dpr10 with the transmembrane domain and C terminus of CD4 (a transmembrane protein not endogenously found in flies). As expected, the cell surface abundance of this chimeric Dpr10-CD4 was unaffected after PLC treatment, suggesting that Dpr10 is anchored at the NMJ via GPI modification (Fig. 5*E*,*F*′,*O*). Conversely, when we replaced the Ig domains of Dpr10 with the last two Ig domains of CD4 but retained the Dpr10 C terminus, this chimeric CD4-Dpr10 was efficiently released from the muscle surface by PLC, demonstrating that the C-terminal portion of Dpr10 indeed contains a GPI anchor (Fig. 5*G*,*H*′,*P*). In experiments with both V5-Dpr10 and CD4-Dpr10, punctate surface labeling was observed after PLC treatment, indicating that some PLC-released Dpr10 was likely retained on the cell surface through its adhesion domain, Ig1, via interactions with endogenous CSPs, including DIP-α. These results demonstrate that Dpr10 and Dpr19 are GPI anchored in their endogenous tissue.

Next, we examined DIP-α. Although DIP-α is not endogenously expressed in muscles (Wang et al., 2022), an ectopically expressed DIP-α variant localized to the SSR (Fig. 5*I*). Like Dpr10 and Dpr19, DIP-α was released from the muscle surface after PLC treatment (Fig. 5*I*,*J*′,*Q*). However, cleavage was less efficient, and DIP-α puncta formed on the muscle surface and around boutons after treatment. Replacing the Ig2 + Ig3 domains and the C terminus of DIP-α with two Ig domains, the transmembrane helix and the C terminus of CD4 blocked the PLC-mediated release from the muscle surface (Fig. 5*K*,*L*′,*R*). Overall, these data show that PLC treatment affects Dpr10, Dpr19, and DIP-α attachment and localization in vivo and support our model that Dprs and DIPs are GPI-anchored CSPs.

### GPI anchoring alters distribution of Dprs and DIPs on neurons and muscles

**Figure 6.**
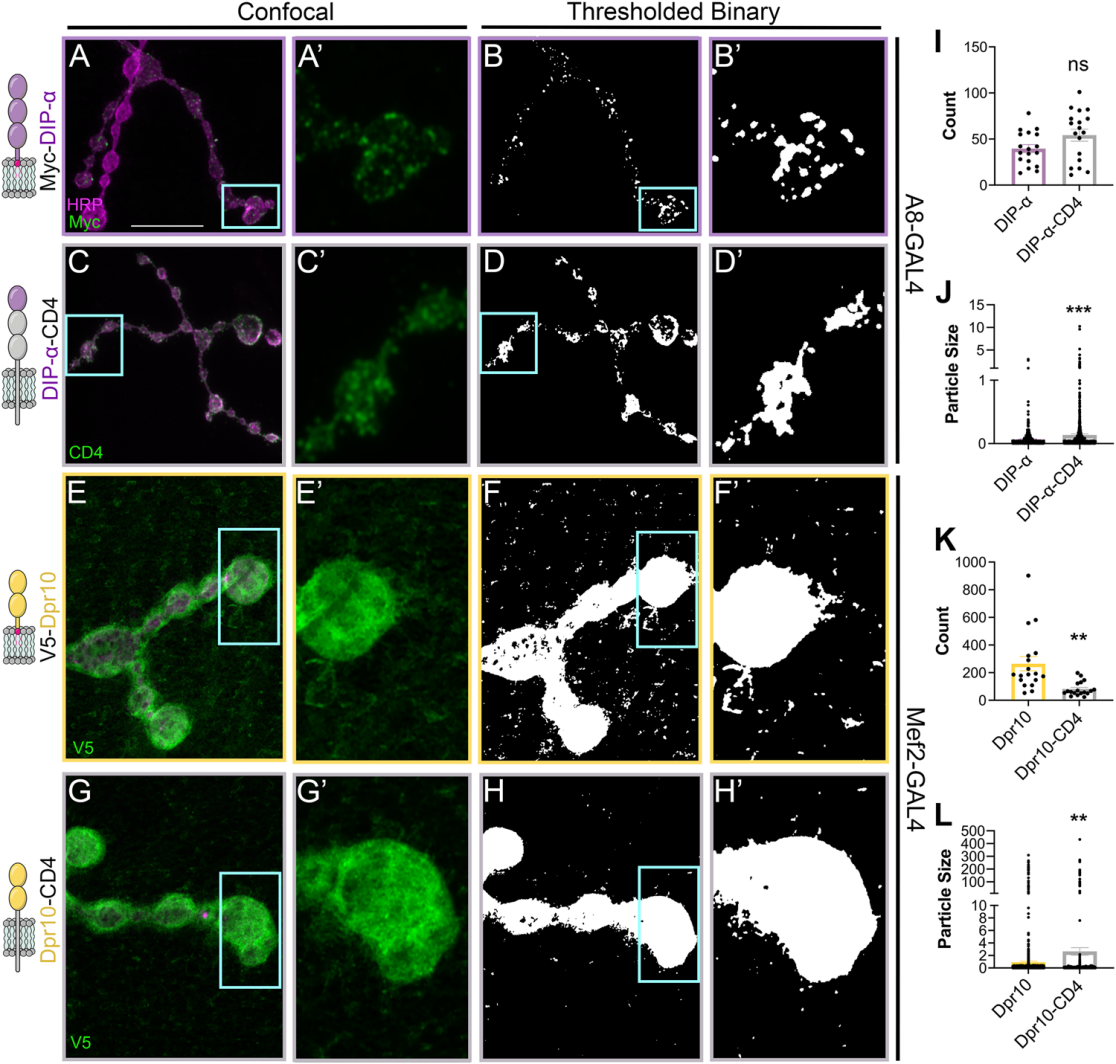
GPI anchors alter distribution of DIP-α on motor neurons and Dpr10 on muscles. ***A–H***′, Diagram depictions of constructs are shown in the left column. Surface localized proteins are shown in green and neuronal tissue in magenta (HRP). Scale bar, 50 µm. ***A***, ***B***, Tagged DIP-α and DIP-α-CD4 were expressed in Is motor neurons. ***A–A***′, *A8-GAL4* driving Myc-DIP-α leads to punctate surface localization on the boutons (*n* = 18/10). ***B–B***′, Binary thresholded image of ***A***. ***C–C***′, *A8-GAL4* driving DIP-α-CD4 leads to larger DIP-α puncta throughout the Is motor neuron terminal (*n *= 18/10). ***D–D***′, Binary thresholded image of ***C***. ***E–E***′, *Mef2-GAL4* driving V5-Dpr10 leads to punctate localization on the muscle surface (*n *= 18/7). ***F–F***′, Binary thresholded image of ***E***. ***G***–***G′***, *Mef2-GAL4* driving Dpr10-CD4 leads to loss of Dpr10 throughout the muscle surface (*n* = 18/7). ***H–H***′, Binary thresholded image of *G*. ***I***, ***K***, Particles counted for experiments depicted in ***A–D*** and ***E–H***, respectively. ***J***, ***L***, Particle sizes for experiments depicted in ***A–D*** and ***E***, ***H***. ns, *p *= 0.0717; **p = 0.0025 in K, *p *= 0.0054 in L; ***p = 0.0002; Welch's *t* test. Processing of thresholded images is further described in Extended Data Figure 6-1.

GPI modifications have been shown to contribute to the subcellular localization of CSPs (Sharma et al., 2004; Arumugam et al., 2021). Thus, we examined localization of ectopically expressed wild-type and chimeric Dpr10 and DIP-α in their endogenous tissues. DIP-α is expressed in a subset of motor neurons called the Is type and localizes to the axon terminals (Carrillo et al., 2015; Ashley et al., 2019; Wang et al., 2022). Using a Is motor neuron-specific driver, *A8-GAL4* (Venkatasubramanian et al., 2019; Wang et al., 2021), we confirmed that Myc-DIP-α localizes to puncta in the motor axon terminal (Fig. 6*A*; Ashley et al., 2019). However, when the GPI anchor was replaced with a TM domain, DIP-α was redistributed into larger puncta, suggesting that proper clustering is partially due to the GPI anchor (Fig. 6*C*). These changes were confirmed by quantifying DIP-α puncta in the terminal three boutons in each condition: in order to determine the nature of this localization difference, samples were thresholded and collapsed to a binary representation (Fig. 6*B*,*D*), and then the number and size of particles in and around the three terminal boutons of each arbor were quantified. Larvae from both conditions were pooled and stained together before images were collected using identical settings, and all samples were thresholded using the same parameters. Myc-DIP-α formed smaller puncta compared with DIP-α-CD4 (Fig. *6I*,*J*; Extended Data Fig. 6-1*A*,*B*), suggesting that the GPI anchor contributes to the subcellular distribution of DIP-α in vivo.

10.1523/ENEURO.0184-23.2023.f6-1 Figure 6-1. **Example particle counting used to examine pre- and postsynaptic localization of Dpr10 and DIP-α.** (A-D) Binary thresholded images; white represents signal above the threshold, black fell below the threshold. Particle count assignment by ImageJ FIJI written in red text above white particles. Cartoons of protein being expressed in lower corner. (A) *A8-GAL4* driving Myc-DIP-α leads to punctate surface localization inside the boutons. (B) *A8-GAL4* driving transmembrane DIP-α-CD4 leads to larger punctate surface labeling of Is arbor. (C) *Mef2-GAL4* driving V5-Dpr10 leads to punctate localization on the muscle surface. (D) *Mef2-GAL4* driving transmembrane Dpr10-CD4 leads to few puncta on the muscle surface. Download Figure 6-1, TIF file.

As described above, expressing Dpr10 in muscles results in Dpr10 localization at the SSR, the membrane folds that form around in presynaptic boutons. However, additional puncta are observed away from boutons on the muscle surface (Fig. 6*E*). When quantifying the muscle field, these puncta were significantly more numerous and smaller when compared with the puncta formed when expressing a chimeric Dpr10 containing a transmembrane helix (Fig. 6*E–H*′,*K*,*L*; Extended Data Fig. 6-1*C*,*D*). Taken together, GPI anchors of DIP-α and Dpr10 contribute to their pre- and postsynaptic membrane distribution, respectively.

## Discussion

Dprs and DIPs have been implicated in many aspects of neural circuit development. However, we lack a systematic analysis of how these CSPs are anchored to the cell membrane to provide insights into their signaling mechanisms. Here, we utilize several in vitro and in vivo approaches to demonstrate that many Dprs and DIPs are attached to the cell membrane through GPI anchors. In tissue, we show that GPI anchors contribute to subcellular clustering on the presynaptic neuronal membrane. Our findings, that Dpr and DIPs are GPI anchored, suggest that Dpr/DIP-mediated functions must be achieved either by merely tethering two cells together and/or by signaling through a coreceptor.

### Predicting GPI anchors

Predicting GPI anchors is intrinsically challenging for multiple reasons. There are no identified consensus sequence motifs for GPI anchor attachment signals. GPI signals are composites of features, including a stretch of hydrophobic amino acids C terminal to the ω residue, chemically resembling transmembrane regions. This results in only ambiguous similarities that can be detected between different GPI-anchored proteins at the sequence level. Therefore, identifying GPI anchor signals from sequence alignments of known GPI-linked proteins is not feasible. In line with this, C termini of Dprs and DIPs are not conserved and cannot be aligned. The GPI prediction software must rely on models that include sequence properties beyond the simple profile searches (Eisenhaber et al., 1999, 1998), and that can be accomplished with methods based on machine learning algorithms like PredGPI (HMM and SVM; Pierleoni et al., 2008) or NetGPI (neural networks; Gíslason et al., 2021). However, the success of these algorithms may depend on the availability of large experimentally validated datasets of GPI anchored proteins, which is known to be lacking (Gíslason et al., 2021). The most recent NetGPI program used here could only use 161 sequences with experimental evidence as its training set. Our data containing 32 new sequences significantly expands this set.

In our predictions, a high number of false negatives were observed, as only one-third of Dprs and DIPs were predicted to be GPI anchored by PredGPI and NetGPI. Conversely, the TM prediction tool Phobius predicted 15 out of 32 Dprs and DIPs and Klingon as TM proteins, suggesting this software may not be robust at discriminating between the two membrane targeting motifs. The newest version of TMHMM, DeepTMHMM, based on a deep learning algorithm, performed significantly better than Phobius, classifying only Dpr9 as a TM protein. Unexpectedly, one-third of Dprs and DIPs were neither predicted to be GPI anchored nor to have a TM helix. Together our findings demonstrate that currently available GPI signal prediction tools can provide useful preliminary insights, especially in combination with TM prediction software. It is likely that significant improvements to predictions will come from larger high-quality training datasets of experimentally determined GPI signal sequences. However, experimental validation may for now be the primary way of assessing GPI anchor presence until better prediction tools are developed.

### Dpr and DIP clustering is aided by their GPI anchors

CSP localization is often critical for their function. Here, we demonstrate that DIP-α, Dpr10, and Dpr19 localized to the postsynaptic membrane when expressed in muscles. Moreover, punctate Dpr10 localization on the muscle surface was diminished when we replaced the GPI anchor with a TM helix suggesting that this modification contributes to its localization. Similarly, DIP-α with a GPI anchor distribute into smaller puncta in presynaptic Is arbors compared with DIP-α with a TM helix. While these data suggest that the GPI anchor contributes to the membrane distribution of DIP-α and Dpr10, we cannot exclude other possibilities. Although expression levels should be nearly identical, as TM and GPI recombinant genes were inserted into the same genomic locus (*DIP-α* and *dpr10* in attP2 and VK20 docking sites, respectively), we noticed that less of the GPI version makes it to the cell surface compared with the TM version. This is likely due to the differential regulation of trafficking of GPI-anchored proteins, which requires additional steps to synthesize and then covalently link the GPI anchor to the protein, before they are trafficked through the secretory pathway separately from TM containing proteins. Thus, it is possible that the membrane distribution observed is, in part, due to the difference of protein levels that make it to the cell surface.

GPI-anchored proteins have been reported to localize to lipid rafts—domains in the lipid bilayer that have increased levels of sterols and are therefore slightly thicker and less mobile than the surrounding membrane (Levental et al., 2020). Although still somewhat controversial, these domains are thought to scaffold proteins by increasing the local concentration of proteins that preferentially localize to lipid rafts such as GPI-anchored proteins, possibly including Dprs and DIPs within these rafts.

The PLC cleavage of Dpr10 and DIP-α from muscles was incomplete, leaving residual punctate signal around the boutons and on the muscle surface unlike Dpr19 that was almost completely lost. These differences in PLC cleavage efficiencies may be due to different local interactions of Dprs and DIPs with various proteins or lipids in the membrane. Alternatively, residual surface labeling could arise following complete cleavage where the cleaved CSPs remain as a result of binding nearby CSPs, preventing release from the tissue.

### Dpr–DIP-mediated cell adhesion depends on their GPI anchors

*Trans*- and *cis*-interactions between Dprs and DIPs drive their roles in the nervous system. Dprs and DIPs may act similar to other cell adhesion molecules (e.g., integrins or cadherins) by clustering to generate avidity and strengthen cell–cell contacts during axonal fasciculation or synapse formation. We examined four Dpr–DIP pairs, and all showed robust aggregation that was susceptible to PLC treatment, suggesting that the GPI anchors in Dprs and DIPs are accessible by PLC at the cell–cell contact sites. This phenomenon could have profound consequences in vivo for modulating cell adhesion, provided that specific lipases and proteases are present under physiological conditions at sufficient concentrations and exhibit high enough substrate turnover to cause structural changes to cell–cell contacts.

Shedding of cell surface molecules by hydrolytic enzymes like proteases and lipases has been implicated in various signaling mechanisms. Examples include activation of fibroblast growth factor 2 (FGF-2) via the release of soluble syndecan-1 (Kato et al., 1998), potentiation of β-catenin signaling upon cleavage of neuronal cadherin (N-cadherin; Reiss et al., 2005), modulation of Nodal signaling via GPI cleavage of CRIPTO (Lee et al., 2016), and bidirectional regulation of the Notch pathway through proteolysis of Notch or its ligands (Mishra-Gorur et al., 2002; Boyer-Di Ponio et al., 2007; Dyczynska et al., 2007; Steinbuck and Winandy, 2018). GPI-anchored proteins are unable to directly signal intracellularly so acting as soluble factors for other receptors, either in *cis* or *trans*, could be a conceivable mode of action. A subset of Dprs (Droujinine et al., 2021) and DIPs were identified in a secretome screen as soluble factors in the hemolymph of larvae (Personal Communication, Norbert Perrimon and Justin Bosch), but the roles of these soluble variants are unknown. In addition to potential functions of soluble forms of these CSPs in the extracellular space, it would be interesting to explore if their shedding in vivo could affect Dpr–DIP-mediated cell adhesion and have consequences for the structure and function of synapses or other specialized sites of cell–cell contact, as shown for some CAMs (Reiss et al., 2005; Rodríguez-Manzaneque et al., 2009; Peixoto et al., 2012; Suzuki et al., 2012; Toth et al., 2013; Stahl et al., 2014).

### GPI anchors in neural circuit assembly

GPI-anchored proteins are critical for many physiological processes, including neural circuit formation. These proteins can be cleaved by lipases or proteases as part of their signaling mechanism. For example, the GPI-anchored protein RECK regulates motor neuron differentiation in mammals by inhibiting Notch signaling; only when RECK is cleaved and released from the membrane can the ADAM10 metalloprotease access and cleave the Notch ligand thereby promoting differentiation (Corda et al., 2014). In addition, IgLONs, the vertebrate orthologs of Dprs and DIPs, are shed from neurons via ADAM10 to promote neuronal growth of nearby neurons (Sanz et al., 2015; Pischedda and Piccoli, 2016). The presence of GPI anchors in Dprs and DIPs suggests that their biology and signaling may be conserved with IgLONs. Finally, if the GPI-anchored protein is not cleaved, it can still signal through a coreceptor that traverses the cell membrane. To our knowledge, no Dpr or DIP coreceptors have been published, and this provides an intriguing future avenue of research.

## Data Availability

All microscopy images and flow cytometry data used in our analyses are available upon request; the processed results are presented here. All remaining data are located in this article.

## Synthesis

Reviewing Editor: Lynda Erskine, University of Aberdeen Decisions are customarily a result of the Reviewing Editor and the peer reviewers coming together and discussing their recommendations until a consensus is reached. When revisions are invited, a fact-based synthesis statement explaining their decision and outlining what is needed to prepare a revision will be listed below. The following reviewer(s) agreed to reveal their identity: Oren Schuldiner.

Your manuscript has been reviewed by 2 experts in the field. Both found your work to be potentially interesting, providing new information relevant to Dpr/DIP proteins as well as the field of cell surface molecules in general. However, there are a number of technical and textual concerns that need to be addressed. This includes, clarifying some aspects of the text, dampening some of the "all or most claims", and some additional experiments. Below is a synthesis of the reviewers' comments.

General comments on the manuscript:

In this manuscript, the authors study the way Dpr/DIPs are attached to the membrane. this has been an outstanding question in the field as most of these proteins do not have clear trasnmembranal domain predictions - in fact, using different algorithms arises in different results. Likewise, algorithms that predict GPI anchors also did not consistently predict specific members of the Dpr/DIP IgSF proteins. Using a variety of in vitro assays, the authors convincingly demonstrate that most, if not all members of the Dpr/DIP family are, or at least can be, GPI anchored proteins. The paper adds important information for the field. However, there are a few technical and textual concerns that the authors should address before publication.

The work in this manuscript systematically characterizes an important but unknown biochemical property of the cell adhesion molecules, DIPs and Dprs. DIPs heterophilically interact with Dprs to regulate neuronal wiring and axon organization, yet whether these proteins are associated with the cell membrane by a transmembrane domain or GPI links is unclear. The authors provide compelling evidence showing most, if not all, of DIPs/Dprs are anchored to the membrane by GPI modifications instead of traversing the lipid bilayer by transmembrane domains. They demonstrate this new finding by cell line-based in vitro assays (computational approaches and PLC treatment), as well as in vivo functional data supporting their findings for select DIP/Dpr members. Furthermore, this manuscript also demonstrates that the GPI anchor is critical to the cell adhesion functions of DIP-Dpr interactions by in vitro cell aggregation assay, and in vivo protein subcellular localization in the larval NMJ. These results suggest the functional importance of GPI anchors of DIPs/Dprs in neuronal wiring and survival. Interestingly, the results of the paper also suggest that many of the cellular functions of DIPs/Dprs also depend on unknown coreceptors. Overall, while the in vivo evidence on how GPI anchor of DIP/Dpr proteins contribute to neuronal functions is relatively preliminary, we appreciate that the major biochemical findings of this paper is timely and valuable to the field and would be of great interest to broader neuroscientists focusing on cellular/developmental neurobiology, cell-cell adhesion, biochemistry/chemical biology, and beyond. There are, however, some concerns/suggestions that need to be addressed.

Major points to be addressed:

1) a critical point which needs to be clarified is where exactly was the V5 tag inserted. I am assuming it was inserted in the N-terminal side, but after the signal peptide. This is not clearly written and is important to understand how the proteins make it to the right place. Ideally, the author should be able to know the exact bp of the V5 insertion.

2) both figure 1-1 and 1C are not optimal and a bit difficult to interpret. Why show the probability of TM domain if it is not in full correlation with the prediction (in 1-1)? Isn't it better to just show a table with the prediction from both algorithms? Similarly, why is PredGPI shown in a quantitative way while NetGPI in a binary analysis? Likewise, It is unclear why the threshold of 99% was chosen for PredGPI and a much lower threshold is chosen (and not shown or discussed) for NetGPI.

3) For the computational prediction, after running transmembrane domain classification, the authors immediately hypothesized non-transmembrane Dprs/DIPs might have GPI anchor. It would be good to provide a more solid rationale for why the authors came up with this hypothesis given that there are other types of membrane anchors that the authors also mention.

4) Would AlphaFold perform better in protein structure prediction? Some recent work shows that AlphaFold at least works nicely for transmembrane structure prediction (see doi: 10.1093/nar/gkac928 and doi: 10.1007/s00018-021-04112-1).

5) The reviewers had complementary suggestions for improving figure 2 as detailed below:

Figure 2 is impressive but difficult to comprehend in its current form. It is impossible to write in the text "Remarkably, all Dprs and DIPs displayed these trends, suggesting that Dprs and DIPs are GPI anchored" - when the figure is not easy to analyze. A quantification - for example, showing the sup/pellet signal for each condition, is a must - especially given some of the not easily interpreted results. Still relevant to figure 2 - the explanation for the doublets is one hypothetical solution, I would be a bit more careful in wordings. Final comment about this figure - I would be a bit more careful with stating that the figure supports that "all or most" of Dpr/DIPs are GPI anchored.

Figure 2 is straightforward but too busy and mostly showing qualitative data. To make the PLC sensitivity of each examined protein more informative, a cumulative quantification graph for the band intensities on the gel is needed that shows results for each DIP and Dpr tested. As some of the supernatant bands with PLC+ appear to be not drastically higher than the PLC- control, such as DIP-ε, including a quantification panel would make it less confusing. The authors may also consider colour coding the protein name based on their relative levels cleaved by PLC.

Since membrane-bound Klingon, which is used as a positive control, was not completely released from the membrane by PLC treatment in Figure 2, it would be better to include another positive control with known GPI anchors (i.e. Sema7A and/or ephrinA5).

6) Figure 3 is very cool. However, it would be a nice proof to now take the proteins for which you have a CD4 version (from figure 5 and 6) and test them for PLC cleavage (figure 2, and figure 3) and for cell aggregation (figure 4).

Also for the flow cytometry assay presented in Figure 3, we recommend adding an antibody isotype (IgG) control. It is possible that PLC treatment may remove the surface components that non-specifically bind to the antibody which may account for the difference between PLC- and PLC+ samples.

7) For cell aggregation assay in Figure 4, do EGFP-expressing cells aggregate with mScarlet-expressing cells in the absence of transgenes carrying DIPs and Dprs? This is an important control that seems to be missing.

8) Figure 5-1 is not productive. What can we learn about DIP-ζ?

9) Several suggestions were made for improving Figure 6:

It is great to see the in vivo data supporting that DIPs/Dprs are GPI-anchored. The authors overexpressed tagged protein in muscles of transgenic animals to provide evidence for this in vivo. As probably authors also know, there are antibodies against the some of the DIPs/Dprs, like DIPα and Dpr10 (Xu S, et al., Neuron 2018), and DIPβ and DIPγ (Xu C, et al, Neuron 2019). It might be useful to test whether these proteins can be released from the cell surface by PLC in vivo directly using these antibodies and bypassing potential issues with protein tag/overexpression?

Figure 6 is the weakest link in this manuscript. Best is to study localization of the endogenous protein (there are techniques established to tag endogenous proteins, even in sparse populations of neurons). I understand this will take too much time and therefore I would strongly suggest to tone down the importance of the results here. In any case - "we confirmed that Myc-DIP-α localizes to puncta in the motor axon terminal" - has anyone shown that the endogenous DIP-α is localized in this fashion? if not, why assume?

Furthermore, both reviewers were not convinced with the changes shown, especially not in E-H. The figure would benefit from higher-resolutions images, for example expansion microscopy coupled with some sort of super resolution (airyscan is ok) should be used to show this presumed redistribution in higher resolution.

Additional points:

1) In introduction section, "axonal pathfinding and fasciculation (Barish et al., 2018; Bornstein et al., 2021; Lobb-Rabe et al., 2022), cell fate determination (Courgeon and Desplan, 2019), cell survival (Carrillo et al., 2015; Xu et al., 2019, 2022), and behavior (Brovero et al., 2021)."

We believe cell survival should also cite "Courgeon and Desplan, 2019; Menon et al., 2019" while Xu et al., 2019 here should be Xu S et al., 2018; behaviour should also cite "Xu C et al., 2019". (There are two Xu scientist in the field so just a reminder to double check. We also recommend the authors check other citation throughout the text.)

2) "Phobius predicted 15 of the Dprs and DIPs and Klingon to have C-terminal TM regions while DeepTMHMM indicated only Dpr9 as a TM protein (Figure 1-1)." What Klingon is should be introduced when it first appears in the text. We are aware that this is a cell surface protein but might not be so familiar to other readers.

3) "Specificity" index in the Figure 1 and the corresponding text is confusing, it sounds like it is suggesting false positive rate? Can the authors just use "probability of the protein to be GPI- anchored"?

4) "we setup an S2 cell culture pipeline with V5-tagged, full-length proteins (see Table 1-1 and the methods section for details)", where V5 first appeared, the authors should specify that V5 is tagged to the N-terminus. This appears later in the text but would make it easier to follow if it was described early on.

5) For Figure 2 and Figure 3, the authors only showed the represented gel blots or flow cytometry graphs. How many biological replicates were performed?

6) For cell aggregation section, "that the expression levels of DIP-β and Dpr21 were comparable to other proteins tested here", I assume the authors are referring to Supplemental Figure 4-1?

7) "Conversely, when we replaced Ig1 of Dpr10 with the first Ig domain of CD4 but retained the second Ig and C-terminus, this chimeric CD4-Dpr10 was efficiently released from the muscle surface by PLC (Figure 5G- H',P), indicating that some PLC-released Dpr10 was likely retained on the cell surface through its adhesion domain, Ig1, via interactions with endogenous Dprs and DIPs." It is hard to follow the logic here, as efficiently releasing CD4-Dpr10(C-terminus) would just suggest Dpr10 does have GPI at its C-terminus, isn't it? Additionally, the authors mentioned that Dpr19 signal is almost lost after PLC treatment while Dpr10 has some portion retained on the surface. This is true from the confocal images, but for the quantification graphs, the levels after PLC treatment are comparable. What is the explanation for this?

8) It would be good to colour code the Dpr10 and CD4 text the same way as their representations in the schematics in both Figures 5 and 6. This would make it significantly easier to follow.

9) "When quantifying the muscle field, these puncta were significantly reduced when compared to the localization of the chimeric DIP-α containing a transmembrane helix (Figure 6K-L; Figure 6-1C-D)" Should this be Dpr10 instead of DIP-α? It should also be compared with the transmembrane version- is there an increase in the number of puncta in the muscle field instead of a reduction?

10) An alternative explanation for the differences in the quantification of puncta between the GPI-anchored version and the transmembrane version might arise from the expression level differences between these transgenes. Are these transgenes inserted at the same genomic locus? The authors should include this information in the fly line table.

11) One intriguing point the authors discuss is that the vertebrate orthologs of Dprs/DIPs, IgLONs, are shed from the cell surface by ADAM10. Can Dprs/DIPs also be cleaved by ADAM proteases, in addition to PLC?

12) We know that this is out of the scope of the paper but are the transgenes/chimeras used in vivo functional? Can they rescue previously generated mutants?

## Author Response

Synthesis Statement for Author:

Your manuscript has been reviewed by 2 experts in the field. Both found your work to be potentially interesting, providing new information relevant to Dpr/DIP proteins as well as the field

of cell surface molecules in general. However, there are a number of technical and textual concerns that need to be addressed. This includes, clarifying some aspects of the text, dampening some of the "all or most claims", and some additional experiments. Below is a synthesis of the reviewers' comments. We agree with many of these comments and implemented them in our revised version. General comments on the manuscript: In this manuscript, the authors study the way Dpr/DIPs are attached to the membrane. this has been an outstanding question in the field as most of these proteins do not have clear transmembranal domain predictions - in fact, using different algorithms arises in different results. Likewise, algorithms that predict GPI anchors also did not consistently predict specific members of the Dpr/DIP IgSF proteins. Using a variety of in vitro assays, the authors convincingly demonstrate that most, if not all members of the Dpr/DIP family are, or at least can be, GPI anchored proteins. The paper adds important information for the field. However, there are a few technical and textual concerns that the authors should address before publication. The work in this manuscript systematically characterizes an important but unknown biochemical property of the cell adhesion molecules, DIPs and Dprs. DIPs heterophilically interact with Dprs to regulate neuronal wiring and axon organization, yet whether these proteins are associated with the cell membrane by a transmembrane domain or GPI links is unclear. The authors provide compelling evidence showing most, if not all, of DIPs/Dprs are anchored to the membrane by GPI modifications instead of traversing the lipid bilayer by transmembrane domains. They demonstrate this new finding by cell line-based in vitro assays (computational approaches and PLC treatment), as well as in vivo functional data supporting their findings for select DIP/Dpr members. Furthermore, this manuscript also demonstrates that the GPI anchor is critical to the cell adhesion functions of DIP-Dpr interactions by in vitro cell aggregation assay, and in vivo protein subcellular localization in the larval NMJ. These results suggest the functional importance of GPI anchors of DIPs/Dprs in neuronal wiring and survival. Interestingly, the results of the paper also suggest that many of the cellular functions of DIPs/Dprs also depend on unknown coreceptors. Overall, while the in vivo evidence on how GPI anchor of DIP/Dpr proteins contribute to neuronal functions is relatively preliminary, we appreciate that the major biochemical findings of this paper is timely and valuable to the field and would be of great interest to broader neuroscientists focusing on cellular/developmental neurobiology, cell-cell adhesion, biochemistry/chemical biology, and beyond. There are, however, some concerns/suggestions that need to be addressed. We appreciate that the reviewers recognized our contribution and address their concerns below. Major points to be addressed: 1) a critical point which needs to be clarified is where exactly was the V5 tag inserted. I am assuming it was inserted in the N-terminal side, but after the signal peptide. This is not clearly written and is important to understand how the proteins make it to the right place. Ideally, the author should be able to know the exact bp of the V5 insertion. This is a very important point and was central to our construct design. The V5 tag had to be placed after the signal peptide on the N-terminal side of the protein, as the reviewer recognized. We added this important detail in the text in additional places. We made sure this is repeated when we describe our constructs for the flow cytometry and cell aggregation assays as well. Since the signal peptide is different for every Dpr and DIP, we cannot state one specific amino 2 acid position for the placement of V5, as the reviewer suggested. Therefore, we include a new Table that has the full sequence of all constructs tested in our PLC assays, where all features are labeled (Table 2-1). 2) both figure 1-1 and 1C are not optimal and a bit difficult to interpret. Why show the probability of TM domain if it is not in full correlation with the prediction (in 1-1)? Isn't it better to just show a table with the prediction from both algorithms? Similarly, why is PredGPI shown in a quantitative way while NetGPI in a binary analysis? Likewise, it is unclear why the threshold of 99% was chosen for PredGPI and a much lower threshold is chosen (and not shown or discussed) for NetGPI. We share the reviewer's frustration. Different software report different scores that are unrelated and cannot be compared with each other. PredGPI reports a specificity index, which indicates GPI anchoring when above 0.99. NetGPI reports a classification, GPI anchored or not, and then reports a likelihood of the ω-site if classified as GPI anchored, or lack of GPI anchoring otherwise. Therefore, it is simply not possible to report confidence levels for these predictions in a consistent way. To make the figures easier to digest, we adjusted both Figures 1C and 1-1 to show the qualitative predictions without the specificity index for GPI predictions by PredGPI or the posterior probability of TM helix in TM domain predictions by Phobius. However, the full information is presented in Table 1-2, where we further describe how to interpret these scores. 3) For the computational prediction, after running transmembrane domain classification, the authors immediately hypothesized non-transmembrane Dprs/DIPs might have GPI anchor. It would be good to provide a more solid rationale for why the authors came up with this hypothesis given that there are other types of membrane anchors that the authors also mention. Our main rationale for the GPI anchor hypothesis stems from the fact that the vertebrate homologs of Dprs and DIPs, IgLONs, were shown to be GPI-anchored. Additional mention of it was included in the Results section to make that more apparent. 4) Would AlphaFold perform better in protein structure prediction? Some recent work shows that AlphaFold at least works nicely for transmembrane structure prediction (see doi: 10.1093/nar/gkac928 and doi: 10.1007/s00018-021-04112-1). We thank the reviewer for the suggestion. As structural biologists, we now heavily use Alphafold predictions for our work. Alphafold excels at predicting secondary structure, e.g., an α-helix. However, Alphafold does not know or report if the helix is transmembrane or not. For the two papers mentioned by the reviewer: The Dobson et al. manuscript assesses where the membrane plane may reside on a known transmembrane protein when modeled by Alphafold, and if the Alphafold model is realistic given where the lipid membrane would reside. Their work does not predict whether a protein is transmembrane. The Hegedűs et al. paper is an assessment of Alphafold predictions for transmembrane proteins given known TM protein folds and properties. Again, their work helps interpret Alphafold models for transmembrane proteins, but does not predict if proteins are transmembrane. Both studies make the point that Alphafold was not trained on physical/chemical properties of proteins or their environments, and therefore cannot report on them. 5) The reviewers had complementary suggestions for improving figure 2 as detailed below: Figure 2 is impressive but difficult to comprehend in its current form. It is impossible to write in the text "Remarkably, all Dprs and DIPs displayed these trends, suggesting that Dprs and DIPs are GPI anchored" - when the figure is not easy to analyze. A quantification - for example, showing the sup/pellet signal for each condition, is a must - especially given some of the not 3 easily interpreted results. Still relevant to figure 2 - the explanation for the doublets is one hypothetical solution, I would be a bit more careful in wordings. Final comment about this figure - I would be a bit more careful with stating that the figure supports that "all or most" of Dpr/DIPs are GPI anchored. We thank the reviewers for this point. To address this, we have added a figure (Supplementary Figure 2-1), displaying the quantification of supernatant and cell fractions in control and treatment conditions. Both panels show the raw integrated densities, a number representing the summed pixels in the region of interest, of samples that did not receive PLC in black and samples that did receive PLC treatment in magenta. The lines link the − and +PLC values obtained from an individual experiment. We have added this figure, a corresponding figure legend, and an updated methods section. In addition, we have softened our language based on the conclusions of this figure. About the doublets: We are not certain about their source, even though previous works using PLC assays have also observed them, and in some cases, identified the source. We clarified the text to mention that while observing these doublets is common, we did not investigate them in our work. Figure 2 is straightforward but too busy and mostly showing qualitative data. To make the PLC sensitivity of each examined protein more informative, a cumulative quantification graph for the band intensities on the gel is needed that shows results for each DIP and Dpr tested. As some of the supernatant bands with PLC+ appear to be not drastically higher than the PLC- control, such as DIP-ε, including a quantification panel would make it less confusing. The authors may also consider colour coding the protein name based on their relative levels cleaved by PLC. We thank the reviewers for this suggestion. We hope that the supplementary figure with quantification (Figure 2-1), discussed above, addresses most of the concerns of this point. We agree that this figure is large and very busy, and that it can be confusing. We spent considerable time optimizing this layout, having tested multiple different versions of this figure. We found that color coding the protein names based on their relative levels of cleavage by PLC made the figure appear even more busy and confusing and have opted to leave the names in black print. We hope that the quantification helps the readers interpret this figure. Since membrane-bound Klingon, which is used as a positive control, was not completely released from the membrane by PLC treatment in Figure 2, it would be better to include another positive control with known GPI anchors (i.e. Sema7A and/or ephrinA5). We used Klingon as a positive control and observed that most of it was removed from cells following PLC treatment (see image to the right à). The original work that identified Klingon as a GPI-anchored protein (Butler, 1997) demonstrated this using the same assay, where cleavage was incomplete (appears <50% removed) when assessed by an SDS-PAGE gel. Based on this, we believe our assay is working well. We also think that Klingon is the proper control for our proteins, since it has a similar domain content, and distantly belongs to the same family of proteins as Dprs and DIPs. We purveyed literature for the same assay, which probes proteins in whole cell lysates on gels, including both cell surface and immature proteins in the secretory pathway. Most studies, including one for Sema7A, showed similarly incomplete PLC activity in this assay (see Figure 2C in PMID: 9712866. SemaK1 has been renamed Sema7A.) Given the amount of work needed to repeat the experiments with another control, we do not think it is necessary to add a second positive control, which is unlikely to perform better. From our

Figure 2B.

4 6) Figure 3 is very cool. However, it would be a nice proof to now take the proteins for which you have a CD4 version (from figure 5 and 6) and test them for PLC cleavage (figure 2, and figure 3) and for cell aggregation (figure 4).

We have now repeated our flow cytometry experiments with Dprs and DIPs modified to have a transmembrane helix attachment. We used Dprs and DIPs where the GPI signal sequences were replaced with the transmembrane domains (TMD) from Neurexin-1. The TMD-anchored versions of Dprs and DIPs showed little to no differences in surface display upon PLC treatment. This is now added as a supplement in Figure 3-1.

Also for the flow cytometry assay presented in Figure 3, we recommend adding an antibody isotype (IgG) control. It is possible that PLC treatment may remove the surface components that non-specifically bind to the antibody which may account for the difference between PLC- and

PLC+ samples.

We have now performed isotype control experiments for flow cytometry. We observed no non specific binding for the isotype antibody. These results are newly presented in Figure 3A-G. and Supplemental Figure 3-1. 7) For cell aggregation assay in Figure 4, do EGFP-expressing cells aggregate with mScarlet expressing cells in the absence of transgenes carrying DIPs and Dprs? This is an important control that seems to be missing.

We have now performed the suggested experiment, where we tested for aggregation between EGFP-only and mScarlet-only expressing Sf9 cells. The experiment revealed no aggregation in the absence of Dprs and DIPs. The results were included in Figures 4E and 4-1. 8) Figure 5-1 is not productive. What can we learn about DIP-ζ? We agree. We have now removed this figure from the manuscript. 9) Several suggestions were made for improving Figure 6: It is great to see the in vivo data supporting that DIPs/Dprs are GPI-anchored. The authors overexpressed tagged protein in muscles of transgenic animals to provide evidence for this in vivo. As probably authors also know, there are antibodies against the some of the DIPs/Dprs, like DIPα and Dpr10 (Xu S, et al., Neuron 2018), and DIPβ and DIPγ (Xu C, et al, Neuron 2019). It might be useful to test whether these proteins can be released from the cell surface by PLC in vivo directly using these antibodies and bypassing potential issues with protein tag/overexpression? We thank the reviewer for the suggestion to test endogenous proteins for PLC cleavage. We have antibodies against the four Dprs/DIPs suggested and tested them at the NMJ. Unfortunately, each antibody displayed high background at the larval neuromuscular junction and across the muscle surface. An example image for DIP-β is included below. Thus, we were unable to use antibodies for this analysis. 5

Figure 6 is the weakest link in this manuscript. Best is to study localization of the endogenous protein (there are techniques established to tag endogenous proteins, even in sparse populations of neurons). I understand this will take too much time and therefore I would strongly suggest to tone down the importance of the results here. In any case - "we confirmed that Myc DIP-α localizes to puncta in the motor axon terminal" - has anyone shown that the endogenous DIP-α is localized in this fashion? if not, why assume? Furthermore, both reviewers were not convinced with the changes shown, especially not in E-H. The figure would benefit from higher-resolutions images, for example expansion microscopy coupled with some sort of super resolution (airyscan is ok) should be used to show this presumed redistribution in higher resolution. We thank the reviewer for the comment and the suggestion to obtain and examine higher resolution images. First, Ashley et al. eLife (2019) showed that an endogenously tagged DIP-α distributes to motor neuron terminals in a puncta pattern. We updated the text and referenced this study to highlight the previous punctate DIP-α localization pattern. Second, the Carrillo lab previously attempted expansion microscopy of the larval neuromuscular junction but was unsuccessful. We are continuing to troubleshoot the protocol, but unfortunately, we were unable to apply it to this analysis. However, to gain higher-resolution distribution of DIP-α and Dpr10, we used Airyscan super-resolution microscopy, as the reviewer suggested, and replaced the images in Figure 6. Indeed, we were able to resolve the puncta more clearly for both GPI and transmembrane variants (see example below).

Old Figure 6A-D:

New Figure 6A-D:

6

Additional points:

1) In introduction section, "axonal pathfinding and fasciculation (Barish et al., 2018; Bornstein et al., 2021; Lobb-Rabe et al., 2022), cell fate determination (Courgeon and Desplan, 2019), cell survival (Carrillo et al., 2015; Xu et al., 2019, 2022), and behavior (Brovero et al., 2021)."

We believe cell survival should also cite "Courgeon and Desplan, 2019; Menon et al., 2019" while Xu et al., 2019 here should be Xu S et al., 2018; behaviour should also cite "Xu C et al., 2019". (There are two Xu scientist in the field so just a reminder to double check. We also recommend the authors check other citation throughout the text.) We have edited and manually modified the citations to further disambiguate author names.

2) "Phobius predicted 15 of the Dprs and DIPs and Klingon to have C-terminal TM regions while DeepTMHMM indicated only Dpr9 as a TM protein (Figure 1-1)." What Klingon is should be introduced when it first appears in the text. We are aware that this is a cell surface protein but might not be so familiar to other readers. Klingon is now introduced when it is first mentioned in the manuscript.

3) "Specificity" index in the Figure 1 and the corresponding text is confusing, it sounds like it is suggesting false positive rate? Can the authors just use "probability of the protein to be GPI anchored"? "Specificity index" is a term introduced by the authors of the PredGPI algorithm, and is not exactly a probability value, but a measure of it. We used it in the previous version of the manuscript to adhere to the terminology from their original paper, but this is clearly confusing.

During the revisions, according to suggestions of the reviewers, we adjusted Figure 1 to no longer include the plot with specificity indices. The specificity index is mentioned in the text in the corresponding Results section and the supplemental Table 1-2, where we explain that it is a measure for the likelihood of the presence of a GPI-anchor, and describe how to interpret it.

4) "we setup an S2 cell culture pipeline with V5-tagged, full-length proteins (see Table 1-1 and the methods section for details)", where V5 first appeared, the authors should specify that V5 is tagged to the N-terminus. This appears later in the text but would make it easier to follow if it was described early on.

The information about the placement of the V5 tag was added at the suggested place in the text, and additionally in other places for extra clarification.

5) For Figure 2 and Figure 3, the authors only showed the represented gel blots or flow cytometry graphs. How many biological replicates were performed? During revisions, we repeated the flow cytometry experiments using five biological replicates, and the plot shown in Figure 3H now includes all the data points.

6) For cell aggregation section, "that the expression levels of DIP-β and Dpr21 were comparable to other proteins tested here", I assume the authors are referring to Supplemental Figure 4-1? That is correct. The supplemental Figure 4-1 is now referenced in the main text with the statement that "both DIP-β and Dpr21 were expressed at considerable levels".

7) "Conversely, when we replaced Ig1 of Dpr10 with the first Ig domain of CD4 but retained the second Ig and C-terminus, this chimeric CD4-Dpr10 was efficiently released from the muscle surface by PLC (Figure 5G- H',P), indicating that some PLC-released Dpr10 was likely retained on the cell surface through its adhesion domain, Ig1, via interactions with endogenous Dprs and DIPs." It is hard to follow the logic here, as efficiently releasing CD4-Dpr10(C-terminus) would just suggest Dpr10 does have GPI at its C-terminus, isn't it? Additionally, the authors mentioned that Dpr19 signal is almost lost after PLC treatment while Dpr10 has some portion retained on 7 the surface. This is true from the confocal images, but for the quantification graphs, the levels after PLC treatment are comparable. What is the explanation for this? We agree with reviewer that this sentence was not clearly articulated and have made adjustments so that it now reads "Conversely, when we replaced the Ig domains of Dpr10 with the last two Ig domains of CD4 but retained the Dpr10 C-terminus, this chimeric CD4-Dpr10 was efficiently released from the muscle surface by PLC, demonstrating that the C-terminal portion of Dpr10 indeed contains a GPI anchor (Figure 5G-H',P). In experiments with both V5-Dpr10 and CD4-Dpr10, punctate surface labeling was observed after PLC treatment, indicating that some PLC-released Dpr10 was likely retained on the cell surface through its adhesion domain, Ig1, via interactions with endogenous CSPs, including DIP-α." [lines 267-273]. Regarding the discrepancy between the images and quantification graphs: Indeed, it seems that the residual Dpr10 after PLC treatment (Figure 5D') is significantly more than the residual Dpr19 (Figure 5B'). However, if one looks more closely at Figure 5B', there is significant Dpr19 distributed across the entire muscle surface compared to Figure 5D' which has residual Dpr10 concentrated at the NMJ and minimal across the rest of the muscle surface. Because the signal was calculated across the image, the levels after PLC treatment are comparable.

8) It would be good to colour code the Dpr10 and CD4 text the same way as their representations in the schematics in both Figures 5 and 6. This would make it significantly easier to follow. We have added some coloring for the Dpr and DIP labels in Figures 5 and 6, but limited it, as it risked being more confusing.

9) "When quantifying the muscle field, these puncta were significantly reduced when compared to the localization of the chimeric DIP-α containing a transmembrane helix (Figure 6K-L; Figure 6-1C-D)" Should this be Dpr10 instead of DIP-α? It should also be compared with the transmembrane version- is there an increase in the number of puncta in the muscle field instead of a reduction? We thank the reviewers for bringing this to our attention. Indeed, we meant to state Dpr10 instead of DIP-α. Also, based on our updated analysis, the Dpr10 variant with the transmembrane helix displayed fewer and larger puncta across the muscle surface compared to the GPI-anchored variant. The sentence is now corrected.

10) An alternative explanation for the differences in the quantification of puncta between the GPI-anchored version and the transmembrane version might arise from the expression level differences between these transgenes. Are these transgenes inserted at the same genomic locus? The authors should include this information in the fly line table. The reviewer is correct that expression level differences could explain the differences in puncta quantification. We have responded to this in text, including a mention for the possibility of differences in expression levels. Also, the transgenes are inserted at the same genetic loci (the DIP-α constructs are in attP2 and the Dpr10 constructs are in VK20). We included this detail in the text. Additionally, we have expanded the Methods section to include more details about how we generated the transgenic UAS lines and included the insertion sites in the table.

11) One intriguing point the authors discuss is that the vertebrate orthologs of Dprs/DIPs, IgLONs, are shed from the cell surface by ADAM10. Can Dprs/DIPs also be cleaved by ADAM proteases, in addition to PLC? We strongly agree with the reviewer on this point. For this, we have purified a soluble variant of Kuzbanian (the Drosophila homolog of ADAM10) and tested for its proteolytic cleavage activity 8 on Sf9 cells expressing a subset of Dprs and DIPs. We assessed levels of proteins in the cell lysates and the cell culture media upon protease treatment using western blots, but observed no differences for the protease-treated samples. While these results are discouraging, our tests have not been exhaustive. It may be worth testing a broader range of reaction conditions, other constructs of Kuzbanian (e.g. the full-length transmembrane protein), other ADAM proteases present in Drosophila (Kuzbanian-like, Meltrin and Tace), and the remaining Dprs and DIPs. These are avenues for future studies.

12) We know that this is out of the scope of the paper but are the transgenes/chimeras used in vivo functional? Can they rescue previously generated mutants? These transgenes/chimeras are currently being used in other ongoing work and is beyond the scope of this study.
