# Supplementary figures and images for: Neuronal Wiring Receptors Dprs and DIPs Are GPI Anchored and This Modification Contributes to Their Cell Surface Organization
Source: eNeuro. 2024 Feb 9;11(2):ENEURO.0184-23.2023. doi: 10.1523/ENEURO.0184-23.2023 (PMC10863630; doi:10.1523/ENEURO.0184-23.2023)

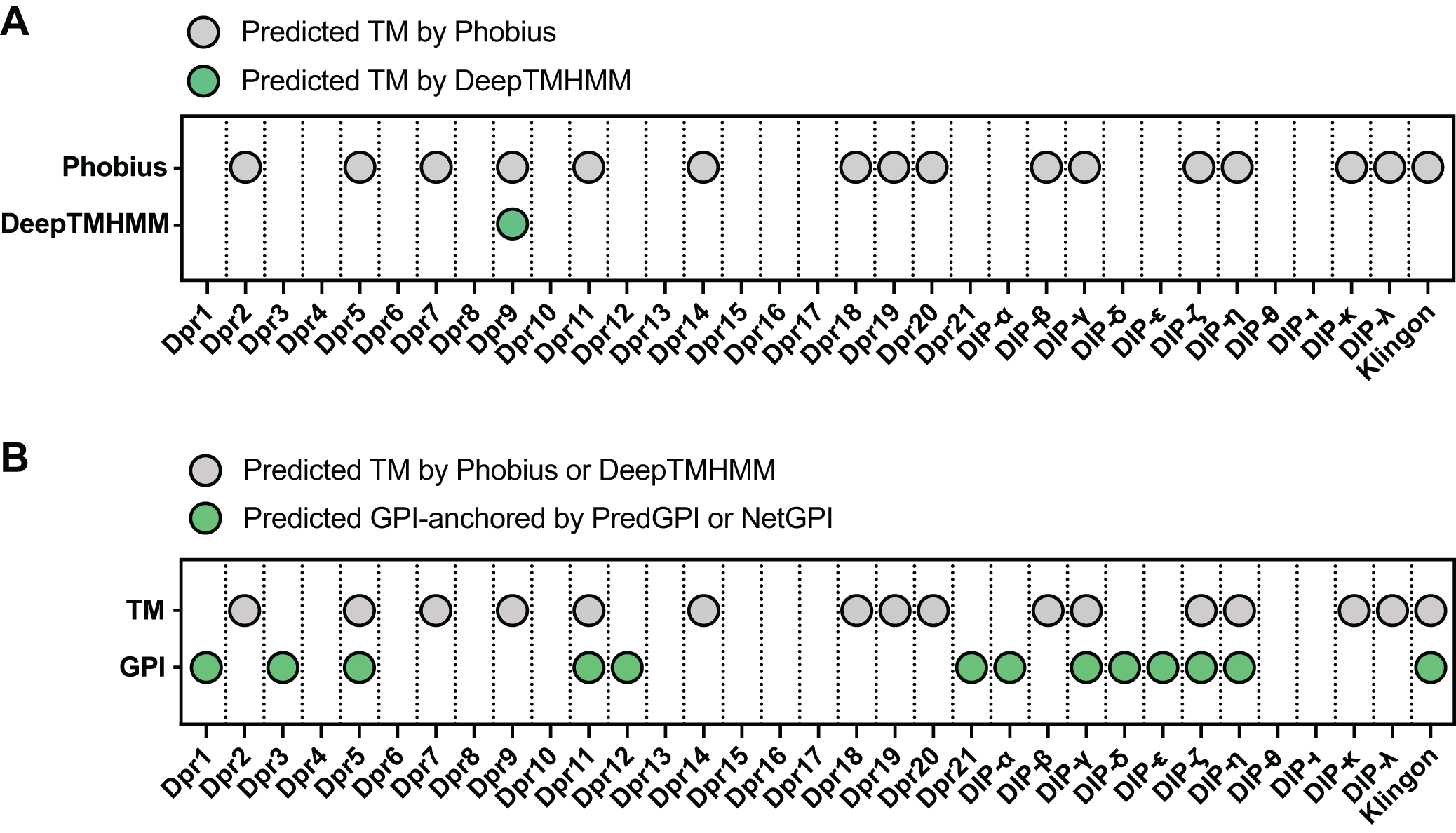

Supplement: Figure 1-1. — Transmembrane region and GPI anchoring predictions. (A) Positive predictions for the presence of transmembrane domains are shown as green circles for DeepTMHMM and gray circles for Phobius. (B) Positive GPI anchoring predictions by at least one program are shown as green circles, and positive TM predictions by at least one program are shown as gray circles. Download Figure 1-1, TIF file. [file eneuro-11-ENEURO.0184-23.2023-s002.tif]

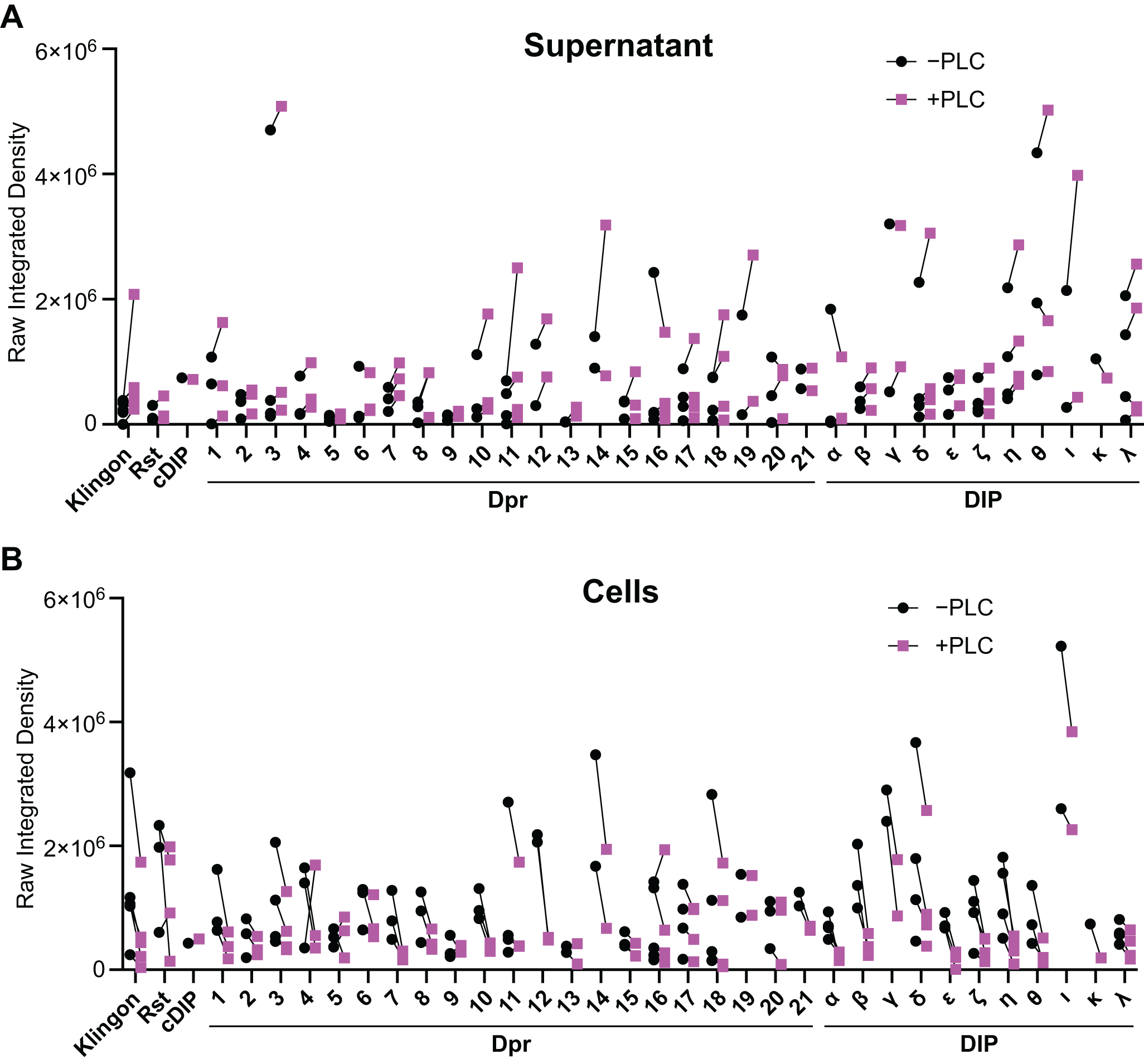

Supplement: Figure 2-1. — Quantification of western blots of PLC treated Dprs and DIPs from S2 cells. (A) Raw integrated density values of western blots from experiments examining the supernatant fraction of Dprs and DIPs expressed in S2 cells (see Figure 2 for example blots). Black circles are values obtained from control samples that were not treated with PLC, while magenta squares are samples that were treated with PLC. Values that come from one experiment are linked with a black line. The protein examined is denoted on the x-axis. (B) Depicts the same experimental quantification but for the cell fraction rather than the supernatant. Download Figure 2-1, TIF file. [file eneuro-11-ENEURO.0184-23.2023-s003.tif]

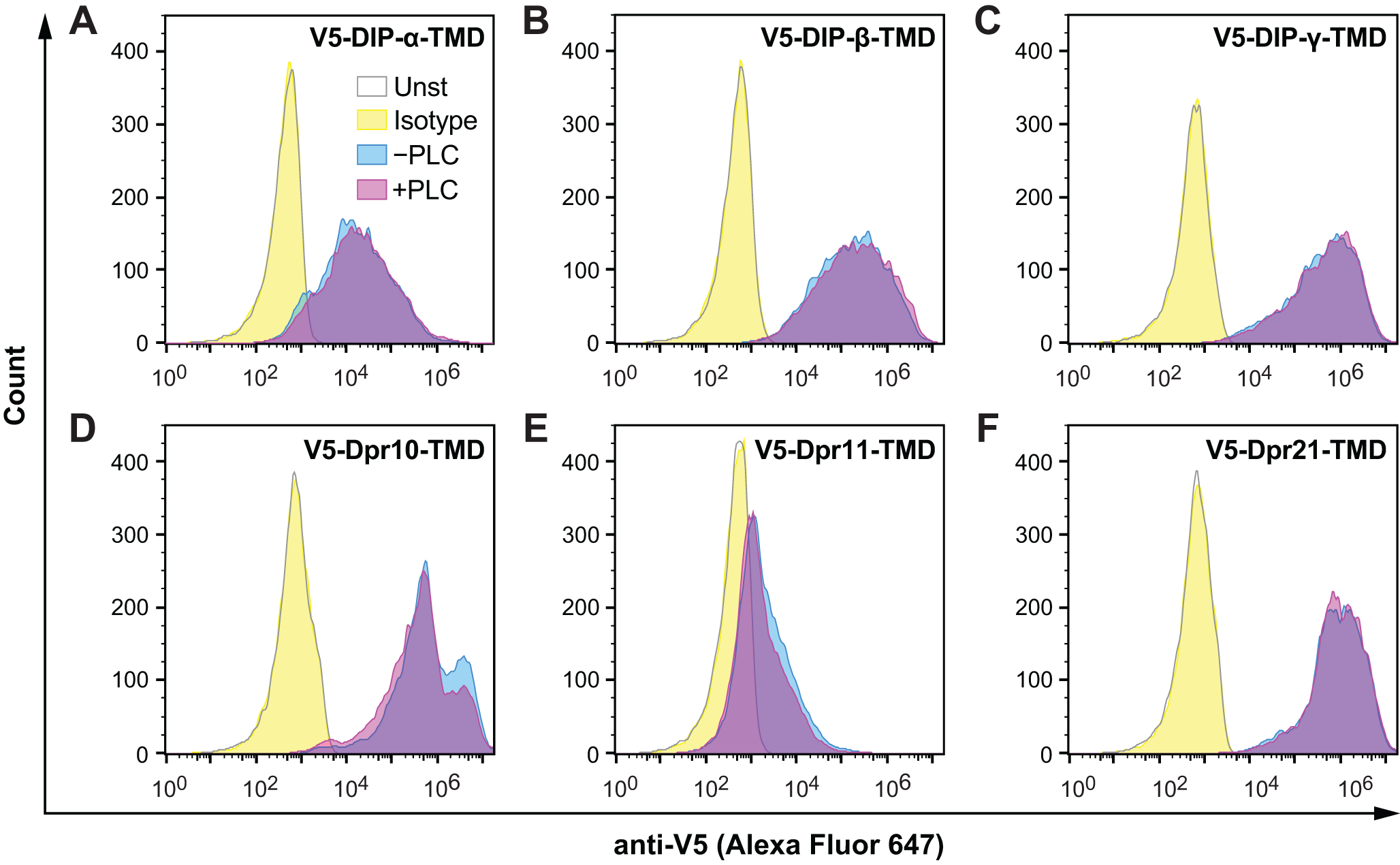

Supplement: Figure 3-1. — Surface display of transmembrane versions of Dprs and DIPs is not affected by PLC treatment, as observed by flow cytometry. (A-F) Histograms showing fluorescence levels of baculovirus-infected, unstained Sf9 cells (gray), cells infected and stained with anti-V5-Alexa Fluor 647 antibody (blue), cells infected, treated with PLC and stained with anti-V5-Alexa Fluor 647 antibody (magenta), and cells infected and stained with rabbit IgG isotype Alexa Fluor 647 antibody (yellow). TMD: transmembrane domain from rat Neurexin-1. Download Figure 3-1, TIF file. [file eneuro-11-ENEURO.0184-23.2023-s004.tif]

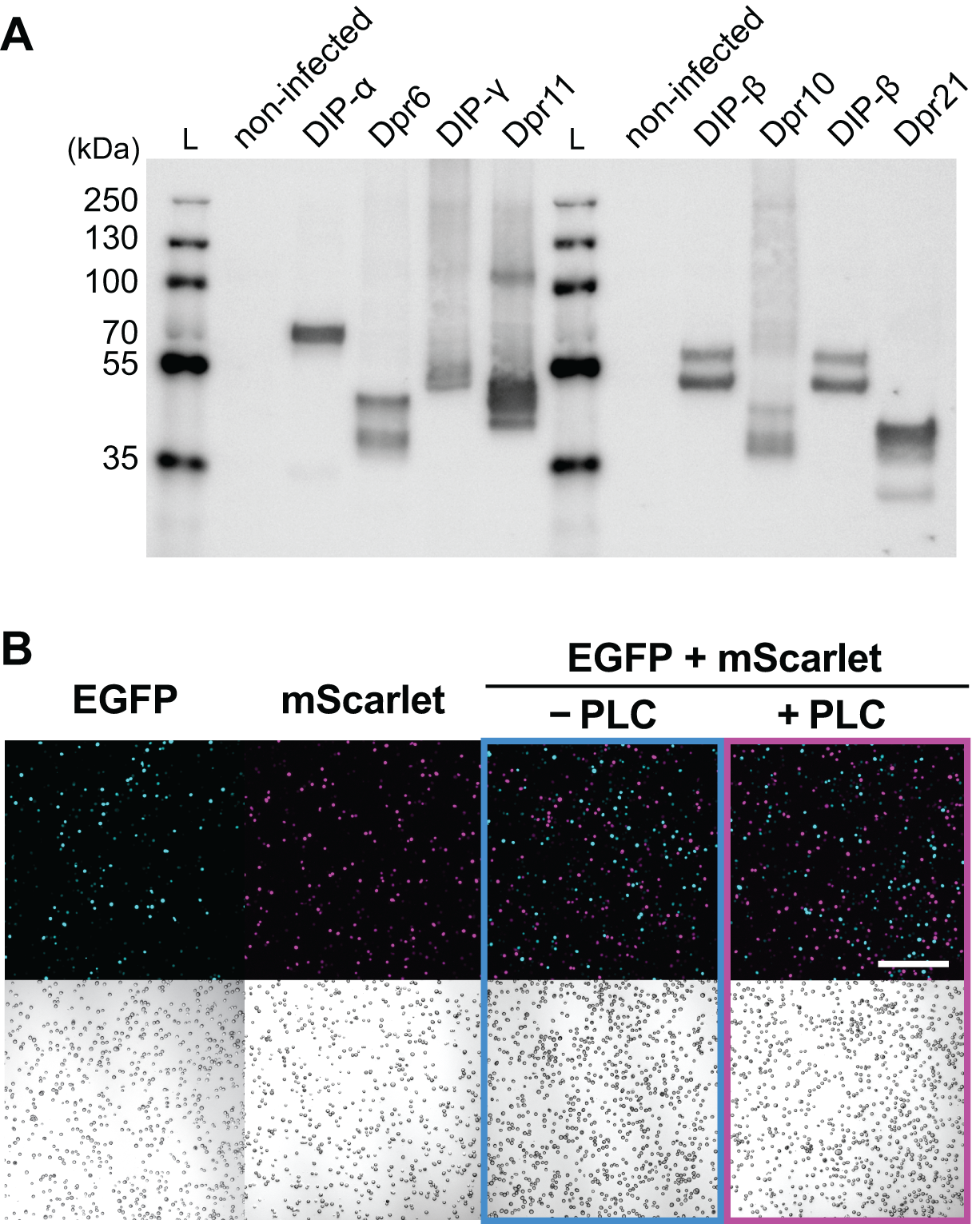

Supplement: Figure 4-1. — (A) Expression of N-terminally V5-tagged Dprs and DIPs in Sf9 cells used in cell aggregation assays. Sf9 cell pellets were solubilized and used in western blot with anti-V5-Alexa Fluor 647 antibody. L, molecular weight ladder. Expected molecular weights of proteins before N-linked glycosylation: DIP-α: 58.7 kDa; Dpr6: 41.9 kDa; DIP-γ: 45.7 kDa; Dpr11: 36.2 kDa; DIP-β: 53.1 kDa; Dpr10: 40.7 kDa; Dpr21: 31.7 kDa. Dprs and DIPs are N-glycosylated to various extent. (B) Negative control for cell aggregation experiments. Cell aggregation assay was performed with Sf9 cells infected with baculoviruses encoding only intracellular fluorescent proteins, EGFP and mScarlet. Scale bar, 500 μm. No aggregation was observed. Download Figure 4-1, TIF file. [file eneuro-11-ENEURO.0184-23.2023-s005.tif]

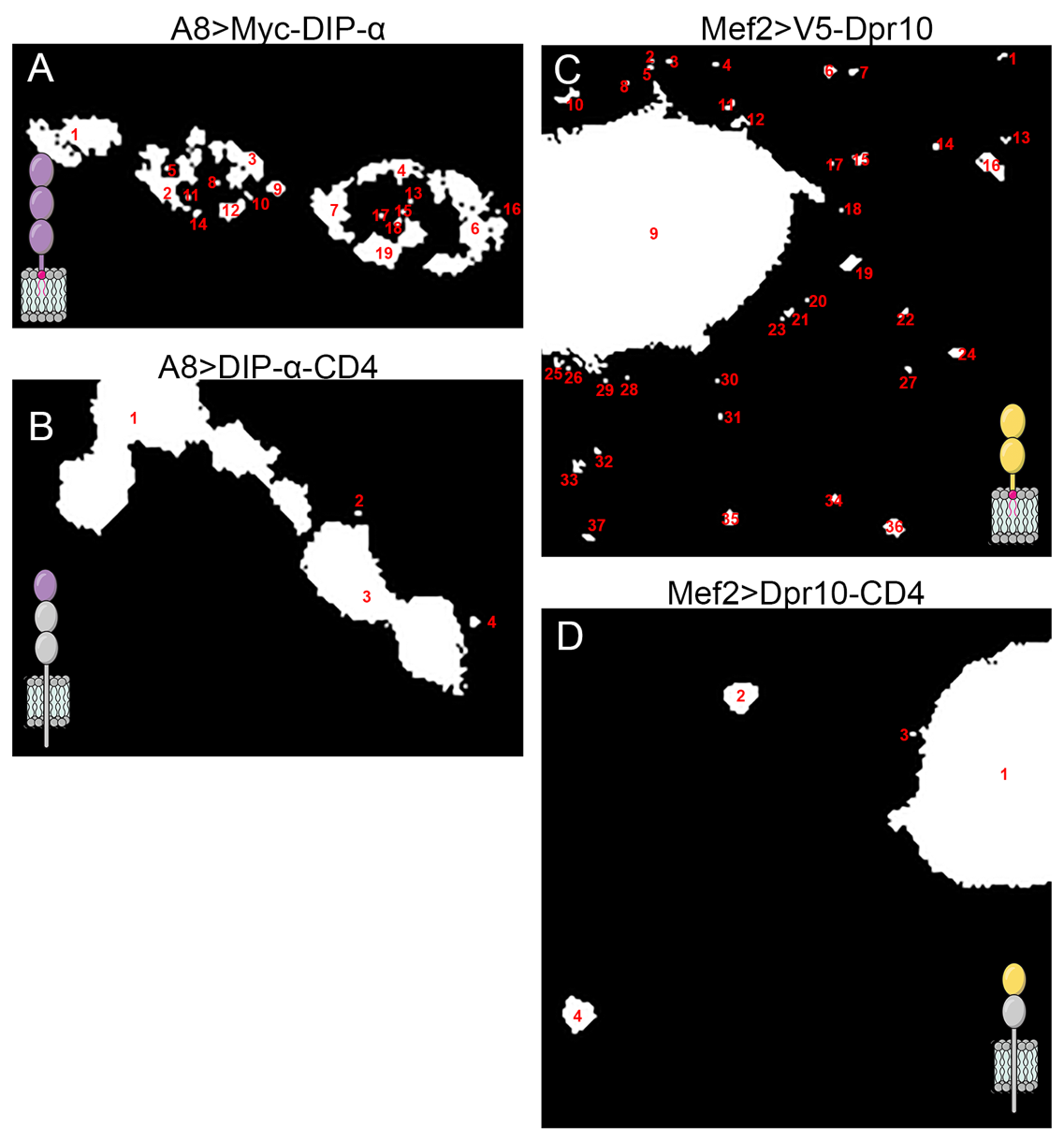

Supplement: Figure 6-1. — Example particle counting used to examine pre- and postsynaptic localization of Dpr10 and DIP-α. (A-D) Binary thresholded images; white represents signal above the threshold, black fell below the threshold. Particle count assignment by ImageJ FIJI written in red text above white particles. Cartoons of protein being expressed in lower corner. (A) A8-GAL4 driving Myc-DIP-α leads to punctate surface localization inside the boutons. (B) A8-GAL4 driving transmembrane DIP-α-CD4 leads to larger punctate surface labeling of Is arbor. (C) Mef2-GAL4 driving V5-Dpr10 leads to punctate localization on the muscle surface. (D) Mef2-GAL4 driving transmembrane Dpr10-CD4 leads to few puncta on the muscle surface. Download Figure 6-1, TIF file. [file eneuro-11-ENEURO.0184-23.2023-s006.tif]
